# Gene expression analysis of nuclear factor I-A deficient mice indicates delayed brain maturation

**DOI:** 10.1186/gb-2007-8-5-r72

**Published:** 2007-05-02

**Authors:** Yong Wee Wong, Christian Schulze, Thomas Streichert, Richard M Gronostajski, Melitta Schachner, Thomas Tilling

**Affiliations:** 1 Zentrum für Molekulare Neurobiologie Hamburg, Universitätsklinikum Hamburg-Eppendorf, Martinistrasse 52, D-20246 Hamburg, Germany; 2 Institut für Klinische Chemie, Universitätsklinikum Hamburg-Eppendorf, Martinistrasse 52, D-20246 Hamburg, Germany; 3 Department of Biochemistry and Program in Neuroscience, State University of New York at Buffalo, 140 Farber Hall, 3435 Main Street, Buffalo, NY 14214, USA; 4 Keck Center for Collaborative Neuroscience and Department of Cell Biology and Neuroscience, Rutgers University, 604 Allison Road, D-251, Piscataway, NJ 08854, USA

## Abstract

**Background:**

Nuclear factor I-A (NFI-A), a phylogenetically conserved transcription/replication protein, plays a crucial role in mouse brain development. Previous studies have shown that disruption of the *Nfia *gene in mice leads to perinatal lethality, corpus callosum agenesis, and hydrocephalus.

**Results:**

To identify potential NFI-A target genes involved in the observed tissue malformations, we analyzed gene expression in brains from *Nfia*^-/- ^and *Nfia*^+/+ ^littermate mice at the mRNA level using oligonucleotide microarrays. In young postnatal animals (postnatal day 16), 356 genes were identified as being differentially regulated, whereas at the late embryonic stage (embryonic day 18) only five dysregulated genes were found. An *in silico *analysis identified phylogenetically conserved NFI binding sites in at least 70 of the differentially regulated genes. Moreover, assignment of gene function showed that marker genes for immature neural cells and neural precursors were expressed at elevated levels in young postnatal *Nfia*^-/- ^mice. In contrast, marker genes for differentiated neural cells were downregulated at this stage. In particular, genes relevant for oligodendrocyte differentiation were affected.

**Conclusion:**

Our findings suggest that brain development, especially oligodendrocyte maturation, is delayed in *Nfia*^-/- ^mice during the early postnatal period, which at least partly accounts for their phenotype. The identification of potential NFI-A target genes in our study should help to elucidate NFI-A dependent transcriptional pathways and contribute to enhanced understanding of this period of brain formation, especially with regard to the function of NFI-A.

## Abstract 2

Gene expression analysis of brains from mice deficient in nuclear factor I-A (*Nfia*^-/- ^mice) and from *Nfia*^+/+ ^mice suggests that *Nfia*^-/- ^mice are delayed in early postnatal development, especially oligodendrocyte maturation.

## Background

The nuclear factor I (NFI) family of sequence-specific DNA binding proteins has four members [1,2] (for review, see Gronostajski [3]), namely NFI-A, NFI-B, NFI-C, and NFI-X. They recognize the nucleotide consensus sequence TTGGC(N)_5_GCCAA. NFI proteins were first identified as nuclear proteins that bind to the replication origin of adenoviruses and initiate DNA replication *in vitro *[4,5]. Their consensus binding sequence was subsequently identified [6-8]. The promoters of several genes were shown to be activated by NFI proteins. These 'positive target genes' include the gene encoding α-globin [9], human hepatitis B virus S gene [10], *Mbp *(myelin basic protein) [11,12], *B-Fabp *(brain fatty acid-binding protein; also called *Blbp *[brain lipid-binding protein]) [13], and *Gabra6 *(α6 subunit of the γ-aminobutyric acid [GABA] type A receptor) [14]. On the other hand, there are also genes that are negatively regulated by NFI, such as the gene that encodes adenine nucleotide translocase 2 [15]. Unpublished data from our laboratory also suggest that NFI-A negatively regulates transcription of the mouse *L1 *gene. L1 is a cell adhesion molecule that is involved in neuronal migration, axon outgrowth, and synaptic plasticity [16]. The complexity of regulation by NFI family members is further increased by alternative splicing, yielding as many as nine different proteins from one gene [17,18]. For instance, a brain-specific isoform of NFI-A [3], which was first isolated in 1990 by Inoue and coworkers [19], activates the transcription of mouse myelin basic protein.

*Nfia*^-/- ^mice exhibit severe neurologic defects, including communicating hydrocephalus, corpus callosum agenesis, and disrupted development of midline glia [20,21], similar to L1-deficient mice [22,23]. These findings indicate that NFI-A plays an important role in regulating gene transcription during brain development. Moreover, NFI-A mRNA is expressed in adult mouse brain [24], which suggests that the respective protein participates in the control of gene expression in the mature central nervous system. To understand how NFI-A could influence brain development and function, it is important to obtain a comprehensive overview of NFI-A responsive genes in the brain. Oligonucleotide microarrays [25] offer an attractive experimental approach for such global gene expression analyses. We therefore performed a microarray analysis of brain cDNA from embryonic (embryonic day 18 [E18]) and early postnatal (postnatal day 16 [P16]) *Nfia*^-/- ^mice in comparison with respective wild-type littermate controls.

Using this method, we identified a large number of genes that are dysregulated at the mRNA level in postnatal NFI-A knockout (*Nfia*^-/-^) mouse brains. Moreover, by *in silico *promoter analysis, we showed that, among this group, at least 70 genes possess phylogenetically conserved NFI binding sites in their promoter region, suggesting that they might be direct NFI-A targets. Database analyses of gene function revealed that the changes in gene expression observed in our study probably reflect a delay in neural, particularly oligodendrocyte, differentiation, which appears to be a consequence of loss of NFI-A.

## Results

### Microarray analysis

High-density oligonucleotide microarray analysis was carried out for total RNA from brains of *Nfia*^-/- ^mice and age-matched, wild-type littermate controls. Analyses were performed with independent samples from three *Nfia*^-/- ^and three wild-type (*Nfia*^+/+^) animals each for E18 and P16. All animals were F_1 _hybrids of C57BL/6 and 129S6 mice, ensuring a survival rate of 38.5% until P16. A total of 356 genes were identified as being differentially expressed in the *Nfia*^-/- ^animals at P16 (197 upregulated and 159 downregulated), taking a cutoff of a 1.2-fold change and a significance of *P *< 0.05 in expression relative to the wild-type control (see Additional data file 1). Among these, 53 genes were found to exhibit a greater than 1.5-fold change in expression (39 downregulated [74%] and 14 upregulated [26%]; Table 1).

**Table 1:** Genes strongly dysregulated in P16 *Nfia*^-/- ^mice

Affymetrix probe set ID	fold change	*P *value	Gene (encoded protein)
92939_at	-3.22	0.0026	*Gabra6 *(γ-aminobutyric acid [GABA]-A receptor, subunit α6)
92940_s_at	-2.94	0.0018	*Gabra6 *(GABA-A receptor, subunit α6)
100068_at	-3.14	0.0075	*Aldh1a1 *(aldehyde dehydrogenase family 1, subfamily A1)
99089_at	-2.43	0.0007	*Mal *(myelin and lymphocyte protein, T-cell differentiation protein)
101887_at	-2.24	0.0007	*Agt *(angiotensinogen precursor, gene, exon 5 and complete coding sequence)
93137_at	-2.21	0.0003	*Ntsr2 *(neurotensin receptor 2)
103023_at	-2.13	0.0003	*Lcat *(lecithin cholesterol acyltransferase)
99046_at	-2.11	0.0080	*Mobp *(myelin-associated oligodendrocytic basic protein)
100536_at	-2.10	0.0048	*Mobp *(myelin-associated oligodendrocytic basic protein)
99047_at	-1.99	0.0011	*Mobp *(myelin-associated oligodendrocytic basic protein)
99048_g_at	-1.98	0.0022	*Mobp *(myelin-associated oligodendrocytic basic protein)
94391_at	-2.07	0.0014	*Gjb6 *(gap junction membrane channel protein β6)
102571_at	-1.81	0.0194	*Gjb6 *(gap junction membrane channel protein β6)
97089_at	-2.06	0.0128	*Folh1 *(folate hydrolase)
160754_at	-1.97	0.0059	*Pygm *(muscle glycogen phosphorylase)
103987_at	-1.95	0.0044	*Mog *(myelin oligodendrocyte glycoprotein)
101467_at	-1.90	0.0018	*S100b *(mouse S100 β protein exons 1 to 3, complete cds.)
100002_at	-1.89	0.0016	*Itih3 *(inter-α trypsin inhibitor, heavy chain 3)
95286_at	-1.88	0.0112	*Clu *(clusterin)
161294_f_at	-1.63	0.0093	*Clu *(clusterin)
100494_at	-1.87	0.0003	*Fgf1 *(fibroblast growth factor 1)
97317_at	-1.81	0.0003	*Enpp2 *(ectonucleotide pyrophosphatase/phosphodiesterase 2)
94144_g_at	-1.71	0.0053	*GFAP *(mouse gene for glial fibrillary acidic protein)
94143_at	-1.56	0.0279	*GFAP *(mouse gene for glial fibrillary acidic protein)
160065_s_at	-1.70	0.0066	*Csrp1 *(cysteine and glycine-rich protein 1)
92608_at	-1.63	0.0194	*Csrp1 *(cysteine and glycine-rich protein 1)
94057_g_at	-1.70	0.0227	*Scd1 *(stearoyl-CoA desaturase; Mouse stearoyl-CoA desaturase gene, exon 6)
94056_at	-1.68	0.0093	*Scd1 *(stearoyl-CoA desaturase; Mouse stearoyl-CoA desaturase gene, exon 6)
161610_at	-1.69	0.0053	*Ndrg2 *(N-myc downstream regulated 2)
96088_at	-1.68	0.0021	*Ndrg2 *(N-myc downstream regulated 2)
94079_at	-1.68	0.0035	*Sept4 *(septin 4)
92717_at	-1.68	0.0135	*Neurod1 *(Neurogenic differentiation 1)
93389_at	-1.67	0.0026	*Prom1 *(prominin 1)
93390_g_at	-1.67	0.0043	*Prom1 *(prominin 1)
98872_at	-1.67	0.0129	*Ugt8 *(UDP-galactose:ceramide galactosyltransferase [Cgt] gene)
93573_at	-1.67	0.0396	*Mt1 *(mouse gene for metallothionein-I [three exons])
96720_f_at	-1.66	0.0145	*Pvalb *(Parvalbumin)
100441_s_at	-1.64	0.0014	*Ank1 *(ankyrin 1, erythroid)
103429_i_at	-1.61	0.0060	(Expressed sequence AL024210)
93750_at	-1.58	0.0011	*Gsn *(murine gelsolin protein; mouse gelsolin gene, complete cds.)
100044_at	-1.58	0.0196	*Cldn11 *(claudin 11)
93159_at	-1.54	0.0065	Expressed sequence tags
92802_s_at	-1.54	0.0333	*Plp1 *(Proteolipid protein [myelin] 1)
92932_at	-1.52	0.0059	*Cbln1 *(cerebellin 1 precursor protein)
102405_at	-1.52	0.0111	*MAG *(myelin-associated glycoprotein)
98338_at	-1.52	0.0363	*En2 *(engrailed 2)
98025_at	-1.51	0.0147	*Evi2a *(ecotropic viral integration site 2a)
93664_at	-1.51	0.0272	*Atp1b2 *(ATPase, Na+/K+ transporting, β2 polypeptide)
102773_at	-1.50	0.0246	*Car8 *(carbonic anhydrase 8)
103046_at	-1.50	0.0079	*Car4 *(*Mus musculus *carbonic anhydrase IV gene, complete cds.)
			
95654_at	1.50	0.0376	*Clic1 *(chloride intracellular channel 1)
92380_r_at	1.58	0.0092	*Ptprz1 *(protein tyrosine phosphatase, receptor type, Z polypeptide 1)
92379_f_at	1.82	0.0020	*Ptprz1 *(protein tyrosine phosphatase, receptor type, Z polypeptide 1)
98394_at	1.58	0.0401	*Dlx1 *(distal-less homeobox 1)
97527_at	1.65	0.0083	*Cks2 *(CDC28 protein kinase regulatory subunit 2)
97520_s_at	1.66	0.0217	*Nnat *(neuronatin)
98038_at	1.72	0.0004	*Hmgb3 *(high mobility group box 3)
101503_at	1.73	0.0339	*Dpysl3 *(dihydropyrimidinase-like 3)
93028_at	1.77	0.0278	*H19 *(H19 fetal liver mRNA)
100600_at	1.84	0.0030	*CD24a *(CD24a antigen)
102307_at	1.87	0.0085	*Dcx *(doublecortin)
101631_at	1.88	0.0169	*Sox11 *(SRY-box containing gene 11)
93669_f_at	2.22	0.0029	*Sox11 *(SRY-box containing gene 11)
93250_r_at	1.92	0.0004	*Hmgb2 *(high mobility group box 2)
96041_at	1.98	0.0037	*Rbm3 *(RNA binding motif protein 3)
98967_at	2.59	0.0002	*Fabp7 *(fatty acid binding protein 7, brain)

Provided is a list of genes significantly dysregulated more than 1.5-fold in *Nfia*^-/- ^mouse brain relative to *Nfia*^+/+ ^mice at postnatal day 16 (P16). Note that several genes (for instance, *Gabra6*) are represented by more than one Affymetrix probe set.

Within this latter group of strongly dysregulated genes, a total of 11 genes exhibit greater than twofold dysregulation, with nine genes downregulated and two upregulated. The downregulated genes include those encoding the following: angiotensinogen (*Agt*); aldehyde dehydrogenase family 1, subfamily A1 (*Aldh1a1*); folate hydrolase (*Folh1*); GABA-A receptor, subunit α6 (*Gabra6*); gap junction membrane channel protein β6 (*Gjb6*); lecithin cholesterol acyltransferase (*Lcat*); myelin and lymphocyte protein (*Mal*); myelin-associated oligodendrocytic basic protein (*Mobp*); and neurotensin receptor 2 (*Ntsr2*). The upregulated genes encode fatty acid binding protein 7 (*FABP7*) and the transcription factor SRY-like HMG-box containing 11 (*Sox11*).

At the late embryonic stage (E18), fewer genes were significantly dysregulated in the *Nfia*^-/- ^mutant relative to the wild-type animals when compared with the postnatal stage (P16). A total of five genes was identified as being significantly dysregulated with changes of more than 1.2-fold (Table 2). One of the three downregulated genes encodes a yet uncharacterized protein, whereas the two others encode phosphatidylinositol-4-phosphate 5-kinase, type II, γ (*Pip5k2c*) and synaptotagmin binding, cytoplasmic RNA interacting protein (*Syncrip*). mRNAs for synaptotagmin 1 (*Syt1*) and pleiomorphic adenoma gene-like 1 (*Plagl1*) were expressed at elevated levels in the *Nfia*^-/- ^animals. At E18, no gene was differentially regulated more than 1.5-fold in the *Nfia*^-/- ^mutants relative to the wild-type controls. Pleiomorphic adenoma gene-like 1 (*Plagl1*) is the only gene that exhibits a 1.2-fold up-regulation in *Nfia*^-/- ^mice at both developmental stages.

**Table 2:** Genes dysregulated in E18 *Nfia*^-/- ^mice

	P16		E18		
		
Affymetrix probe set ID	Fold change	*P *value	Fold change	*P *value	Gene
161763_r_at			-1.40	0.0169	*Pip5k2c *(Phosphatidylinositol-4-phosphate 5-kinase, type II, gamma)
161190_r_at			-1.30	0.00398	RIKEN cDNA 1110057K04 gene
96375_at			-1.23	0.0407	*Syncrip *(Synaptotagmin binding, cytoplasmic RNA interacting protein)
92502_at	1.26	0.00919	1.23	0.023	*Plagl1 *(Pleiomorphic adenoma gene-like 1)
93005_at			1.29	0.00633	*Syt1 *(Synaptotagmin 1)

Provided is a list of genes that are significantly changed at least 1.2-fold in *Nfia*^-/- ^mouse brain relative to *Nfia*^+/+ ^mice at embryonic day 18 (E18). P16, postnatal day 16.

In P16 *Nfia*^-/- ^mice, a total of 356 individual genes, represented by 395 probe sets, were dysregulated in comparison with the wild-type control group. Among these, 35 genes were represented by more than one probe set on the microarray (Additional data file 1, blue labels). In all cases, probe sets representing the same gene showed the same direction of dysregulation when comparing *Nfia*^+/+ ^with *Nfia*^-/- ^mice. For instance, all of the four probe sets for *Mobp *identified a weaker signal in *Nfia*^-/- ^mice than in *Nfia*^+/+ ^mice. Moreover, it is likely that at least four probe sets represent more than one transcript (Additional data file 1, red labels). Therefore, the number of dysregulated genes in *Nfia*^-/- ^mice could even be higher than 356.

**Figure 1:**
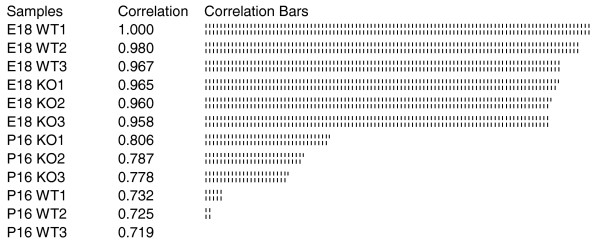
Relative comparison of the individual Genechip results. E18WT1 was used as a template for finding chips with a similar expression profile, using GeneSpring software. All samples were subjected to the correlation comparison. The result shows that the similarity of expression profiles to embryonic day (E)18 *Nfia*^+/+ ^is as follows: postnatal day (P)16 *Nfia*^+/+ ^< P16 *Nfia*^-/- ^< E18 *Nfia*^-/- ^< E18 *Nfia*^+/+^. The E18 *Nfia*^+/+ ^expression profile exhibited greater correlation to the expression profile of P16 *Nfia*^-/- ^than to that for P16 *Nfia*^+/+^. KO, knockout (*Nfia*^-/-^); WT, wild-type (*Nfia*^+/+^).

To investigate overall gene expression profiles, microarray data were analyzed using the robust multi-array average algorithm [26]. Correlations between the expression profiles of individual samples were calculated with all samples, using one *Nfia*^+/+ ^E18 mouse brain ('E18WT1') as a reference for this search (Figure 1). As expected, the greatest similarity in mRNA levels was found between E18WT1 and the two other *Nfia*^+/+ ^mice at E18, namely E18WT2 and E18WT3. Broadly speaking, similarity in the gene expression profile relative to E18WT1 increased in the following order: P16 *Nfia*^+/+ ^< P16 *Nfia*^-/- ^< E18 *Nfia*^-/- ^< E18 *Nfia*^+/+^.

**Figure 2:**
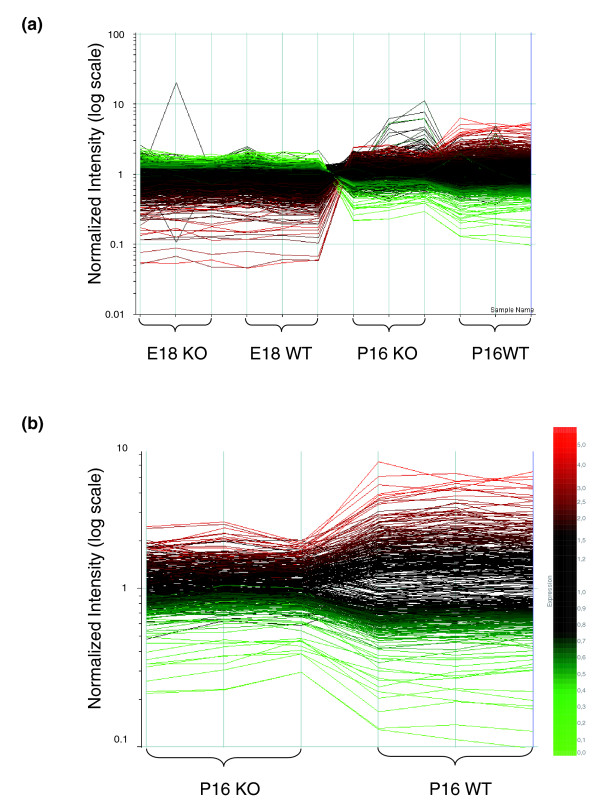
Overall gene expression level in both E18 and P16 *Nfia*^+/+ ^and *Nfia*^-/- ^mice. **(a) **All probe sets. **(b) **The 395 probe sets significantly changed in postnatal day (P)16 *Nfia*^-/- ^relative to P16 *Nfia*^+/+ ^samples. Each curve represents one probe set, and each intercept on the x-axis represents one chip. Two normalization steps were performed. First, normalization across the whole array was carried out in order to correct for variations of average signal intensity. Second, the mean signal intensity of each individual probe set on all 12 chips was set to 1. Taking the rightmost chip on the x-axis ('P16WT3') as a reference (blue line), colors were assigned to the curves representing probe sets. The higher the signal intensity is on this reference chip, the more red the color; similarly, and the lower the signal intensity, the more green is the curve's color (following the spectrum given on the right). KO, knockout (*Nfia*^-/-^); WT, wild-type (*Nfia*^+/+^).

Figure 2a shows the overall gene expression pattern in both E18 *Nfia*^+/+ ^and *Nfia*^-/- ^mice. The expression patterns are much more similar between *Nfia*^+/+ ^and *Nfia*^-/- ^mice than those seen at P16, and very few genes were changed significantly between *Nfia*^+/+ ^and *Nfia*^-/- ^in the E18 animals. In contrast, at P16 many changes in gene expression levels are observed (Figure 2a) between *Nfia*^+/+ ^and *Nfia*^-/- ^animals. Most genes that are expressed at a higher level in E18 become less strongly expressed in P16 mice and *vice versa*, both in *Nfia*^+/+ ^and *Nfia*^-/- ^mice. However, comparison of P16 *Nfia*^-/- ^with P16 *Nfia*^+/+ ^animals revealed many significant changes in gene expression (Figure 2b).

### Gene function

**Figure 3:**
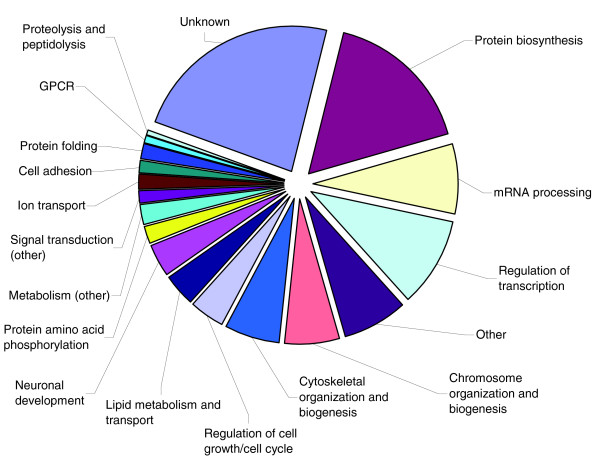
Distribution of gene function among upregulated genes in P16 *Nfia*^-/- ^mice. GPCR, G-protein-coupled receptor signaling; P16, postnatal day 16.

**Figure 4:**
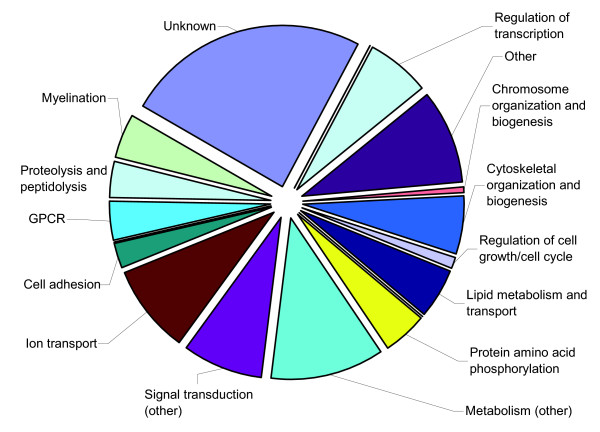
Distribution of gene function among downregulated genes in P16 *Nfia*^-/- ^mice. GPCR, G-protein-coupled receptor signaling; P16, postnatal day 16.

A total of 356 genes that were dysregulated in P16 *Nfia*^-/- ^mice were assigned to biologic functions based on Gene Ontology (GO) Biological Process categories (Additional data file 2 provides a list of assignments). Among the 197 upregulated genes, a large percentage (34.6%) of the candidate genes is involved in transcriptional and translational regulation. These groups include genes that encode RNA binding proteins, transcription factors and ribosomal proteins, and comprise only 10.4% of probe sets on the microarray. Figures 3 and 4 show the distribution of gene functions in the upregulated and downregulated groups, respectively. Within the group of upregulated genes, 33 probe sets (16.8%) were in the category of 'protein biosynthesis', as compared with 1.5% of probe sets on the complete microarray. Twenty probe sets (10.2%) could be assigned to 'regulation of transcription, DNA dependent' (8.2% on the complete microarray), and 15 gene products (7.6%) are involved in mRNA processing (0.7% on the complete microarray). Among the 159 downregulated genes, a significant effect on ion transport related genes can be observed; 14 genes (9.0% of downregulated genes in *Nfia*^-/- ^mice) fall into this category, which is an 'over-representation' compared with the complete array, in which ion transport related genes account for only 1.6% of probe sets.

**Figure 5:**
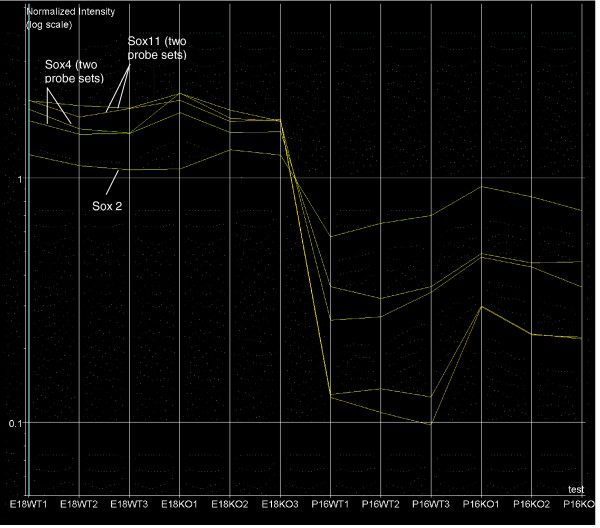
mRNA expression levels of *Sox2*, *Sox4*, and *Sox11*. Shown are mRNA expression levels of the oligodendrocyte precursor genes *Sox2*, *Sox4*, and *Sox11 *in embryonic day (E)18 and postnatal day (P)16 *Nfia*^-/- ^and *Nfia*^+/+ ^mice according to microarray analysis. The line graphs of signal intensities demonstrate that expression levels of these genes decrease from E18 to P16 in both genotypes, but that the reduction in expression is less pronounced in *Nfia*^-/- ^animals. KO, knockout (*Nfia*^-/-^); WT, wild-type (*Nfia*^+/+^).

Interestingly, a number of genes encoding myelin-related proteins exhibited significantly reduced expression in brains of *Nfia*^-/- ^mice at P16 (Figure 4). Because central nervous system myelin is formed by oligodendrocytes, we suspected that NFI-A could influence oligodendrocyte differentiation. We therefore interrogated our list of dysregulated genes for markers of either mature oligodendrocytes or immature oligodendrocyte precursor cells. We found that five genes typically expressed in oligodendrocyte precursors exhibited a higher expression level in *Nfia*^-/- ^mice than in *Nfia*^+/+ ^animals, whereas eight genes that are markers of mature oligodendrocytes exhibited decreased expression in *Nfia*^-/- ^mouse brains (Table 3). As shown in Figure 5, the oligodendrocyte precursor markers *Sox2*, *Sox4*, and *Sox11 *are expressed in both *Nfia*^+/+ ^and *Nfia*^-/- ^animals at E18. However, the decrease in gene expression between E18 and P16 is less pronounced in *Nfia*^-/- ^animals than in *Nfia*^+/+ ^animals, causing an apparent mRNA overexpression of these genes at P16.

**Table 3:** Genes related to oligodendrocyte differentiation are differentially expressed in *Nfia*^-/- ^mice at P16

Gene name	Fold change of mRNA expression in *Nfia*^-/- ^versus *Nfia*^+/+ ^mice^a^	Reference
Genes typically expressed in oligodendrocyte precursors or related to de-differentiation of precursor cells		
*Hmgb2*	1.92	[45]
*Sox2*	1.28	[46]
*Sox4*	1.33/1.44	[47]
*Sox11*	1.88/2.22	[47]
*Tenascin-C*	1.47	[48]
*Myef2*^b^	1.2	[39]
Genes typically expressed in mature oligodendrocytes or related to terminal oligodendrocyte differentiation		
*Mobp*	-2.11/-2.10/-1.99/-1.98	[49]
*Mal*	-2.43	[50]
*Mog*	-1.95	[51]
*Claudin 11*	-1.67	[52]
*Plp1*	-1.58	[53]
*Ugt8*	-1.54/-1.45	[50]
*Mag*	-1.52	[53]
*Dio2*^c^	-1.43	[38]
*Car2*	-1.35	[54]

^a^According to microarray analysis; for genes represented by more than one probe set, the individual fold changes for each probe set are given. ^b^Indirect link to oligodendrocyte maturation (My-EF2 can repress expression of myelin basic protein). ^c^Indirect link to oligodendrocyte maturation (Dio2 catalyzes thyroxine to tri-iodothyronine conversion, and tri-iodothyronine triggers terminal differentiation of oligodendrocytes). P16, postnatal day 16.

Moreover, agenesis of the corpus callosum in animals lacking NFI-A suggests that this transcription factor plays a role in regulating axonal growth. It is tempting to assume that NFI-A could do so by influencing the expression of genes that encode growth promoting or growth repelling proteins. For this reason, we also attempted to identify molecules that have already been shown to stimulate or inhibit neurite growth among those differentially expressed in *Nfia*^-/- ^mouse brains. For 22 genes that were either upregulated or downregulated in the NFI-A mutant animals, reports from the literature indicate that the respective gene product is involved in regulating neurite growth (Table 4). Among these genes, 12 encode proteins whose expression is favorable for neurite growth (for instance acidic fibroblast growth factor (aFGF), melanoma cell adhesion molecule (MCAM) and neural cell adhesion molecule (NCAM), whereas five encode proteins that function in axon repulsion (for example, Ephrin B2 and collapsin response mediator protein-1 (CRMP1)). Five genes encode proteins that influence neurite growth in a cell type or presentation dependent manner, including tenascin-C and CD24.

**Table 4:** Genes encoding modulators of neurite growth are differentially expressed in *Nfia*^-/- ^mouse brains at P16

Gene name	Fold change in mRNA expression in *Nfia*^-/- ^versus *Nfia*^+/+ ^mice^a^	Reference
Genes encoding proteins involved in promotion of neurite growth		
*Clusterin*	-1.88/-1.63	[55]
*S100beta*	-1.90	[56]
*Fgf1 (aFgf)*	-1.87	[57]
*Ndrg2*	-1.69/-1.68	[58]
*Metallothionein 1*	-1.67	[59]
*Gelsolin*	-1.58	[60]
*Metallothionein 2*	-1.49	[59]
*Mcam (gicerin)*	-1.33	[61]
*Basp-1 *(*Cap23*, *Nap22*)	1.24	[62]
*Ncam*	1.29	[63]
*Marcks*	1.32	[64]
*Gap-43*	1.38	[62]
Genes encoding proteins involved in repulsion of neurite growth		
*Brevican*	-1.35	[65]
*Agrin*	1.23	[66]
*EphA5*	1.24	[67]
*Ephrin B2*	1.25	[68]
*Crmp1*	1.43	[69]
Genes encoding proteins which can either be outgrowth-promoting or outgrowth-repelling		
*Mag*	-1.52	[70]
*Caveolin1*	-1.25	[71]
*Sfrp1*	1.28	[72]
*Tenascin-C*	1.47	[73]
*CD24*	1.84	[16]

^a^According to microarray analysis; for genes represented by more than one probe set, the individual fold changes for each probe set are given.

### Quantitative real-time PCR validation of microarray results

**Figure 6:**
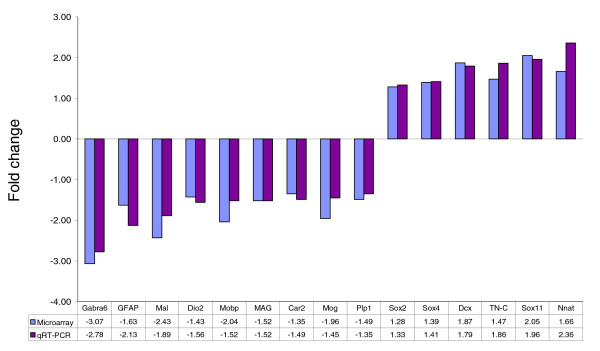
Validation of microarray data by qRT-PCR. The mean fold changes of mRNA expression in postnatal day (P)16 *Nfia*^-/- ^relative to *Nfia*^+/+ ^mice are given on the y-axis. Numbers of independent samples: *n *= 3 for microarray analysis of *Nfia*^-/- ^and *Nfia*^+/+ ^each; *n *= 8 for quantitative real-time polymerase chain reaction (qRT-PCR) analysis of *Nfia*^-/-^; and *n *= 6 for qRT-PCR analysis of *Nfia*^+/+^. Samples used for qRT-PCR include those used in the microarray investigation. Adjacent bars show the microarray and qRT-PCR results for the respective gene. For all 15 genes investigated, the microarray and the qRT-PCR results follow the same direction (either upregulation or downregulation). All differences observed by qRT-PCR are statistically significant (*P *< 0.001 for *Gabra6*, *GFAP*, *Mal*, *Dio2*, *Mobp*, *Sox11*, and *Nnat*; *P *< 0.01 for *MAG*, *Mog*, *Sox2*, *Sox4*, *Dcx*, and *TN-C*; and *P *< 0.05 for *Car2 *and *Plp1*). *Car2*, carbonic anhydrase 2; *Dcx*, doublecortin; *Dio2*, iodothyronine deiodinase II; *Gabra6*, α6 subunit of γ-aminobutyric acid type A receptor; *GFAP*, glial fibrillary acidic protein; *MAG*, myelin-associated glycoprotein; *Mal*, myelin and lymphocyte protein; *Mobp*, myelin oligodendrocyte basic protein; *Mog*, myelin oligodendrocyte glycoprotein; *Nnat*, neuronatin; *Plp1*, proteolipid protein (myelin) 1; *Sox2*, SRY-box containing gene 2; *Sox4*, SRY-box containing gene 4; *Sox11*, SRY-box containing gene 11; *TN-C*, tenascin-C.

Quantitative real-time polymerase chain reaction (qRT-PCR) was performed on 15 genes, selected according to their biologic relevance from the dysregulated genes in P16 *Nfia*^-/- ^mice (Figure 6). On the one hand we chose oligodendrocyte precursor genes such as *Sox2 *and *Sox11*, but we also selected markers of mature oligodendrocytes, such as *Car2 *(carbonic anhydrase 2) and *Mobp *(myelin oligodendrocyte basic protein). In addition to genes relevant to oligodendroglial differentiation, we also chose further markers of immature (*Dcx *[doublecortin]) or mature (*Gfap *[glial fibrilliary acidic protein] and *Gabra6*) neural cells. All PCR analyses were performed in triplicate, using eight independent *Nfia*^-/- ^and six independent *Nfia*^+/+ ^brain samples, and confirmed differential expression of all the genes selected for qRT-PCR validation, indicating the significance of our microarray analysis findings (Figure 6). Importantly, both genes exhibiting a strongly differential expression pattern on the microarrays (for instance, *Dcx*, which exhibited 1.87-fold upregulation) and genes with a much lower fold change (such as *Sox2*, which was 1.28-fold upregulated) were confirmed to be differentially expressed.

**Figure 7:**
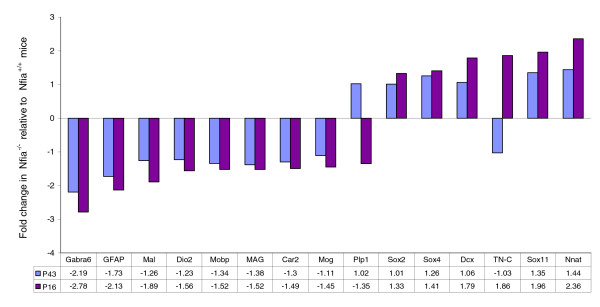
qRT-PCR analysis of gene expression: P43 *Nfia*^-/- ^versus P43 *Nfia*^+/+^. Shown are the findings of quantitative real-time polymerase chain reaction (qRT-PCR) analysis of gene expression in postnatal day (P)43 *Nfia*^-/- ^mice relative to P43 *Nfia*^+/+ ^littermates, in comparison with the respective values for P16 animals. The mean fold changes of mRNA expression in *Nfia*^-/- ^relative to *Nfia*^+/+ ^mice are given on the y-axis. Numbers of independent samples: *n *= 4 for analysis of P43 *Nfia*^-/-^; *n *= 2 for P43 *Nfia*^+/+^; *n *= 8 for P16 *Nfia*^-/-^; and *n *= 6 for P16 *Nfia*^+/+^. Adjacent bars show the P16 and P43 results for the respective gene. *Car2*, carbonic anhydrase 2; *Dcx*, doublecortin; *Dio2*, iodothyronine deiodinase II; *Gabra6*, α6 subunit of γ-aminobutyric acid type A receptor; *GFAP*, glial fibrillary acidic protein; *MAG*, myelin-associated glycoprotein; *Mal*, myelin and lymphocyte protein; *Mobp*, myelin oligodendrocyte basic protein; *Mog*, myelin oligodendrocyte glycoprotein; *Nnat*, neuronatin; *Plp1*, proteolipid protein (myelin) 1; *Sox2*, SRY-box containing gene 2; *Sox4*, SRY-box containing gene 4; *Sox11*, SRY-box containing gene 11; *TN-C*, tenascin-C.

To investigate whether the differential gene expression observed at P16 is maintained at a later age, we also analyzed RNA from postnatal day 43 (P43) *Nfia*^-/- ^and *Nfia*^+/+ ^brains for expression of the selected genes mentioned above. As shown in Figure 7, differences in gene expression between *Nfia*^-/- ^and *Nfia*^+/+ ^animals generally decrease from P16 to P43. However, certain genes such as *Gabra6 *and *Gfap *exhibit pronounced downregulation at both ages.

### *In silico *promoter analysis

NFI-A is a nuclear, DNA-binding protein. It plays a role in adenovirus DNA replication and in transcription of viral and cellular genes. Therefore, we assumed that at least some of the dysregulated genes found in our study might be direct transcriptional targets of NFI-A. In order to identify such potential targets, we conducted a promoter analysis of all genes exhibiting a significant decrease or increase in transcript level in *Nfia*^-/- ^relative to *Nfia*^+/+ ^mice. This *in silico *analysis aimed to detect potential NFI-A binding sites within 2 kilobases (kb) upstream of the respective gene's transcription start site. The palindromic nucleotide sequence TTGGC(N)_5_GCCAA has been demonstrated to be the optimal binding motif for members of the NFI family. However, most of the NFI binding sites experimentally identified thus far do not contain the complete motif, and even half sites can be physiologically relevant [3]. This reflects the fact that transcription factors have a certain degree of freedom in their sequence recognition. For this reason, matrices are used that give the different nucleotides various weightings depending on their importance for transcription factor binding [27]. In addition to the use of such a matrix for NFI binding motifs, we also considered the phylogenetic conservation of these binding sites by comparing the mouse, rat, and human orthologs of the respective genes. We supposed that motifs with a high degree of interspecies conservation are those that are most likely to have physiologic relevance.

Using these criteria, we were able to identify more than 70 genes among our microarray candidate molecules bearing a conserved NFI recognition site in their promoter region (Table 5 and Additional data file 3). This group of genes includes *Gfap *and *Gabra6*, whose promoter activity can be regulated by NFI proteins, according to previous studies [14,28,29]. Interestingly, according to our analysis, *Ncam*, *Vcam1*, *Mcam *and *Mag*, four genes that encode adhesion molecules of the immunoglobulin superfamily, also possess conserved NFI motifs in their promoter sequences.

**Table 5:** *In silico *promoter analysis of genes differentially expressed in *Nfia*^-/- ^mice

Affymetrix probe set ID	Fold change at P16	*P *value	Gene symbol	Number of motifs
100068_at	-3.14	0.01	*Aldh1a1*	3
92642_at	-1.35	0.01	*Car2*	1
103046_at	-1.50	0.01	*Car4*	1
104383_at	1.43	0.00	*Crmp1*	3
102307_at	1.87	0.01	*Dcx*	4
103438_at	-1.43	0.01	*Dio2*	1
98967_at	2.59	0.00	*Fabp7*	1
100494_at	-1.87	0.00	*Fgf1*	1
92940_s_at	-2.94	0.00	*Gabra6*	1
92939_at	-3.22	0.00	*Gabra6*	1
94143_at	-1.56	0.03	*Gfap*	3
94144_g_at	-1.71	0.01	*Gfap*	3
102020_at	-1.22	0.04	*Kcnk3*	2
102405_at	-1.52	0.01	*Mag*	1
96865_at	1.32	0.00	*Marcks*	1
160458_at	-1.33	0.01	*Mcam*	2
100153_at	1.29	0.02	*Ncam*	1
97520_s_at	1.66	0.02	*Nnat*	1
93081_at	1.20	0.03	*Rbbp7*	2
94056_at	-1.68	0.01	*Scd1*	3
94057_g_at	-1.70	0.02	*Scd1*	3
93669_f_at	2.22	0.00	*Sox11*	2
101631_at	1.88	0.02	*Sox11*	2
101993_at	1.47	0.02	*Tnc*	1

Selected genes dysregulated in *Nfia*^-/- ^mice containing one or more conserved NFI recognition motifs within 2 kilobases upstream of their transcription start site. A complete list of all NFI motif carrying genes identified in our analysis is given in Additional data file 2. Note that some genes, such as *Gabra6 *and *Gfap*, were represented by more than one probe set on the microarray. P16, postnatal day 16.

## Discussion

Mice with a targeted ablation of the site-specific transcription factor NFI-A exhibit severe brain malformations, including hydrocephalus and agenesis of the corpus callosum, as was also seen in L1-deficient mice and humans bearing mutations in their *L1 *gene [30]. Most probably, lack of NFI-A causes changes in brain gene expression. Altered expression of genes that encode proteins relevant to brain development might then lead to the observed defects. Therefore, large-scale analysis of mRNA levels in *Nfia*^-/- ^mice could help not only to identify new target genes of NFI-A but also to clarify mechanisms by which this transcription factor influences brain development. For this reason, we used oligonucleotide microarrays to gain gene expression profiles of *Nfia*^-/- ^mouse brains and of corresponding wild-type samples. Relatively early and late changes in gene expression were measured by quantifying transcript levels in *Nfia*^-/- ^and *Nfia*^+/+ ^mice at E18 (before gross hydrocephalus) and at P16 when all animals are clearly hydrocephalic. At P16 stage, we observed that 356 genes were dysregulated in *Nfia*^-/- ^mice relative to *Nfia*^+/+ ^mice. By contrast, only five genes exhibited altered expression in E18 *Nfia*^-/- ^animals in comparison with *Nfia*^+/+ ^mice. Overall, P16 *Nfia*^-/- ^gene expression profiles were more similar to the E18 *Nfia*^+/+ ^than to the P16 *Nfia*^+/+ ^profiles. Hence, one can conclude that *Nfia*^-/- ^mice exhibit a delay in early postnatal brain development relative to wild-type control animals. This idea gains further support when one looks at the function of the dysregulated genes.

### Gene function

When the list of genes changed at P16 was analyzed using Gene Ontology (GO) terms, a total of 34.6% of all upregulated genes in *Nfia*^-/- ^mice fell into the functional groups of transcriptional and translational activities. This is a significantly higher percentage compared with the representation of these groups on the complete microarray (10.4%). In particular, many transcripts encoding ribosomal proteins exhibit elevated levels in these mutants. This upregulation of messages encoding ribosomal proteins suggests increased translational activity in *Nfia*^-/- ^brains.

Interestingly, the expression of several genes associated with immature stages of the nervous system is upregulated in postnatal *Nfia*^-/- ^mice. In particular, elevated mRNA levels of *Dcx *(which encodes doublecortin) and *Nnat *(encoding neuronatin) were observed. Doublecortin is expressed primarily in migrating and differentiating neurons during embryonic development [31]. It is essential for cortical layer formation, most probably because of its role in neuronal migration [32]. Neuronatin is strongly expressed in late fetal and early postnatal brain, but it disappears at later developmental stages [33]. Remarkably, *Plagl1*, the only gene expressed at elevated levels both at E18 and P16 in *Nfia*^-/- ^mice, encodes a transcription factor synthesized preferentially by neural precursor cells [34]. On the contrary, mRNAs for *Gabra6 *and *Gfap*, genes associated with terminal differentiation of neural cells, are found at lower levels in NFI-A deficient mice at P16. The α6 subunit of the GABA-A receptor (encoded by *Gabra6*) is expressed by differentiated neurons in the cerebellum [14]. Like *Gabra6*, *Gfap *(which encodes the glial fibrillary acidic protein, expressed by differentiated astrocytes in the central nervous system) has been identified as a direct target of NFI-A [29] and is downregulated in *Nfia*^-/- ^mice.

The observed pattern of changes in gene expression suggests a delay in brain development in NFI-A mutants. In the absence of NFI-A, genes that are normally expressed during embryonic development and around birth remain at high levels of expression, leading to an overexpression at P16. By contrast, genes whose expression usually increases during the course of terminal differentiation after birth appear not to be activated adequately in *Nfia*^-/- ^mice, leading to their reduced mRNA level in the P16 mutants. Investigation of *Nfia*^-/- ^and *Nfia*^+/+ ^animals at P43 showed that, for most of the selected genes, dysregulation decreased in comparison with P16 or even disappeared. This observation further supports the idea of a delayed expression program in *Nfia*^-/- ^mice.

### Oligodendrocyte differentiation

To identify cell types that are probably affected by the expression delay suggested above, we examined further the function of dysregulated genes. A significant number of myelin-related proteins exhibited decreased expression in *Nfia*^-/- ^mouse brains, prompting us to analyze our results with regard to oligodendrocyte differentiation. Oligodendrocytes produce central nervous system myelin, thereby facilitating rapid impulse conduction [35]. Myelinating oligodendrocytes mainly accumulate after birth in rodents, whereas their progenitors are already apparent in the ventricular zone at around embryonic day 12.5 (E12.5) [36]. Thus, in E18 brains oligodendrocyte progenitor cells are predominant, whereas at P16 mature oligodendrocytes have developed. In *Nfia*^-/- ^brains at P16, we observed that several genes expressed by mature oligodendrocytes, namely *MAG*, *Mal*, *Mobp*, *Mog*, *Ugt8*, *Cldn11*, *Plp1*, and *Car2*, exhibited reduced transcript levels compared to *Nfia*^+/+ ^animals. In contrast, mRNAs encoding Sox2, Sox4, Sox11, tenascin-C and Hmgb2, which are typically expressed by precursor cells rather than by mature oligodendrocytes, are upregulated in *Nfia*^-/- ^brains relative to *Nfia*^+/+ ^brains at this stage. Moreover, *Dio2 *exhibits reduced expression levels in P16 *Nfia*^-/- ^brains. *Dio2 *encodes the iodothyronine deiodinase II, which catalyzes the conversion of the hormone thyroxine to tri-iodothyronine [37]. Tri-iodothyronine is known to trigger terminal oligodendrocyte differentiation [38], and so a reduction in Dio2 levels could delay oligodendrocyte maturation indirectly via reduced tri-iodothyronine levels. Furthermore, we detected slightly increased transcript levels of *Myef2 *in *Nfia*^-/- ^mice. My-EF2, the corresponding protein, represses expression of myelin basic protein [39], a major component of myelin [36,40]. Higher My-EF2 levels could therefore slow down differentiation of oligodendroglia. Moreover, the tremor exhibited by rare NFI-A deficient mice surviving until adulthood [20] would be in accordance with a myelin compromised phenotype. Interestingly, L1 has also been implicated in myelination [41]. Although we did not observe a dysregulation of L1 in our microarray analysis, one cannot exclude an induction in glial cells being obscured by the high expression level of L1 in neurons. In contrast to the peripheral nervous system, glial cells of the central nervous system do not express L1 at any age investigated.

**Figure 8:**
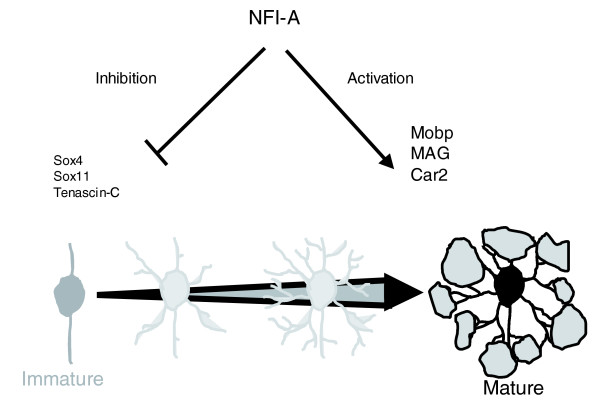
Hypothetical model illustrating how NFI-A could promote oligodendrocyte differentiation. According to this model, nuclear factor I-A (NFI-A) suppresses expression of genes related to oligodendrocyte precursor cells and activates expression of genes whose products are important for mature oligodendrocyte function. For reasons of clarity, only selected genes are indicated.

To summarize, our data suggest a role for NFI-A in regulating terminal differentiation of oligodendrocytes, both by repressing expression of progenitor specific gene products and by enhancing expression of genes that are relevant to mature oligodendrocyte function (Figure 8). It is noteworthy that the time window between E18 and P16, during which these changes in expression pattern emerge, fits nicely to the main period of oligodendrocyte differentiation. In agreement with the observations presented here, a crucial role for NFI-A in spinal cord gliogenesis was recently shown [42], which lends further support to the idea that NFI-A mediates oligodendrocyte differentiation.

### Axonal growth and guidance

The absence of the corpus callosum in *Nfia*^-/- ^mice suggests that NFI-A could participate in controlling expression of molecules relevant for axonal growth and guidance. For this reason, we analyzed whether dysregulated genes detected in our microarray study had been reported to be involved in these processes. For at least 22 of the genes differentially expressed in *Nfia*^-/- ^brains, previous studies indicated a participation of their gene products in growth and guidance of neuronal processes. This is quite a large number, which strengthens the view that NFI-A could contribute to brain wiring during the early postnatal period by regulating the transcription of genes that encode neurite growth promoting or inhibiting proteins. A more detailed look at the dysregulated genes reveals that growth-promoting molecules, such as clusterin, aFGF, and Ndrg2, are expressed at a rather lower level in *Nfia*^-/- ^mice, whereas an upregulation of the repulsive guidance cues such as ephrin B2 and CRMP1 can be observed. However, there are both upregulated growth promoting and downregulated growth inhibiting molecules. Moreover, some molecules can either favor or inhibit growth, depending on their interaction partners or on the way in which they are presented by neurons and glial cells. Therefore, it will be necessary to study NFI-A dependent expression of these proteins at the cellular level in order to shed more light on the role played by NFI-A in axonal growth and guidance.

### Promoter analysis

Because NFI-A is a transcription factor, we assumed that it regulates the transcription of at least some of the differentially expressed genes found in our study. In order to identify such potential targets, we performed an *in silico *analysis of potential, phylogenetically conserved NFI-A binding sites within 2 kb upstream of the respective genes' transcription start sites. We could identify more than 70 genes among our microarray candidate molecules bearing a conserved NFI recognition site in their promoter. Because of their high identity to the NFI consensus sequence combined with phylogenetic conservation, these motifs are likely candidates for transcriptional regulation by NFI-A. Consistent with this assumption, two established direct target genes of NFI-A, *Gfap *[29] and *Gabra6 *[14], were among the candidates detected in our promoter analysis. Two possible reasons for why only about 70 genes carrying conserved NFI motifs were identified from more than 300 genes dysregulated in total are as follows.

First, NFI-A might alter the expression levels of other transcription factors that affect target gene expression (indirect influence). For example, expression of the transcription factor NeuroD1, which is downregulated 1.68-fold in *Nfia*^-/- ^mice, can be increased by GABAergic excitation [43]. This effect is mediated via GABA-A receptors, and *Gabra6*, the gene encoding the α6 subunit of the GABA-A receptor, is a direct target of NFI-A [14], which is in accordance with the results of our promoter analysis. Thus, it is tempting to speculate that the reduced expression of this GABA-A receptor subunit in NFI-A deficient mouse brains could contribute to the observed reduction in NeuroD1 expression.

Second, our promoter analysis probably does not yield a complete list of direct NFI-A targets within the group of dysregulated genes investigated. The limitation to the 2 kb region upstream of the transcription start excludes all potential NFI sites that might be located either further upstream or within the first intron, and even NFI half sites, which are not considered by our stringent screening method, can mediate NFI protein binding under certain conditions. Therefore, the number of direct NFI-A targets among the differentially expressed genes might be even higher than 70.

Most importantly, however, this promoter screen provides a molecular basis for the role of NFI-A in regulating brain maturation; several genes associated with maturation of neurons or glial cells are not only dysregulated in postnatal *Nfia*^-/- ^mice, but also possess NFI binding sites in their promoter, enabling direct transcriptional regulation by NFI-A. Among them, there are both markers of oligodendrocyte precursors (*Sox11 *and *Tenascin-C*) and genes expressed by mature oligodendrocytes (*Car2 *and *Mag*). Therefore, it is likely that NFI-A exerts its influence on postnatal brain development at least partly by directly regulating the transcription of the genes identified in our promoter analysis.

## Conclusion

In the present study, we investigated the effects of NFI-A ablation on brain mRNA expression patterns in mice, aiming to discover potential NFI-A targets that are important for perinatal and early postnatal brain development. Using microarray analysis, only five genes appear to be dysregulated at the transcript level in late embryonic *Nfia*^-/- ^mice, whereas a much higher number, specifically 356, of dysregulated genes was observed at postnatal day 16. We confirmed the changes in expression of 15 genes by qRT-PCR in both the original RNA samples used on the microarrays and in independent samples. Upregulation of immature neural cell markers and downregulation of mature neural cell markers in 16-day-old *Nfia*^-/- ^brains suggest that removal of NFI-A causes a delay in early postnatal brain development. In particular, genes relevant to oligodendrocyte maturation are affected at the crucial time window for this process. An *in silico *promoter analysis revealed that more than 70 dysregulated genes possess a phylogenetically conserved NFI binding site within 2 kb upstream of their transcription start point, including several genes that are implicated in differentiation of oligodendrocytes, astrocytes, and neurons. Therefore, our results suggest that NFI-A plays an important role in early postnatal brain maturation and identify promising potential target genes. Future investigations based on our data should enhance understanding of the processes that govern postnatal development of brain structures.

## Materials and methods

### Animals

*Nfia*^-/- ^mice were bred and maintained as described previously [20]. Animals were killed by exposing them to CO_2 _at embryonic stage E18.5 (E18), postnatal day 16 (P16), or postnatal day 43 (P43). Brains were dissected and snap frozen in liquid nitrogen. The respective *Nfia*^-/- ^and *Nfia*^+/+ ^animals used for analysis at E18, P16, or P43 were littermates. All of the animals were F_1 _hybrids of C57BL/6 and 129S6 animals. In the initial studies of the *Nfia*^- ^allele in a mixed 129S6-C57BL/6-Black Swiss background, we found that about 80% of *Nfia*^-/- ^mice died at P0 and about 10% survived to P16. In contrast, 0% of *Nfia*^-/- ^mice survive past P0 in an inbred C57BL/6 background, precluding studies of postnatal animals. In the inbred 129S6 background about 30% of *Nfia*^-/- ^mice survive to P16, whereas in F_1 _hybrids of C57/BL6 males and 129S6 females about 60% of *Nfia*^-/- ^mice survive to P16. Many of these animals die after weaning from their mothers at P24 and the remainder are severely runted (Gronostajski RM, unpublished observations). Thus, we chose P16 129S6-C57BL/6 F_1 _hybrid animals for analysis because they could be obtained in sufficient numbers, and the *Nfia*^-/- ^mice were genetically identical to their *Nfia*^+/+ ^littermates. Within these matings, males had a B6 and females had a 129S6 background. In a large scale test, 38.5% of the offspring survive until P30 using this type of mating. All animal use was performed under the approved protocol BCH05082N (RMG) of the University at Buffalo Institutional Animal Care and Use Committee in the UB Laboratory Animal Facility, an facility licensed by the Association for the Assessment and Accreditation of Laboratory Animal Care.

### RNA preparation

Total RNA was extracted from the entire brains of *Nfia*^-/- ^and *Nfia*^+/+ ^mice using TRIzol (Invitrogen, Karlsruhe, Germany), in accordance with the manufacturer's instructions. Further purification of RNA was performed using the RNeasy Mini or Midi Kits (Qiagen, Hilden, Germany). Total RNA concentration was determined using a spectrophotometer at 260 nm and 280 nm wavelength. To check RNA integrity, total RNA was separated in a 1% agarose gel containing formaldehyde, and the intensity ratio of 28S and 18S ribosomal RNA bands was assessed after ethidium bromide staining.

### Microarray hybridization and signal detection

Procedures for cDNA synthesis, labeling, and hybridization were carried out in accordance with the manufacturer's protocol (Affymetrix Inc., Santa Clara, CA, USA). All experiments were performed using Affymetrix mouse genome Genechip U74A version 2. Briefly, 15 μg total RNA was used for first-strand cDNA synthesis with a high-performance liquid chromatography purified T_7_-(dT)_24 _primer. Synthesis of biotin-labeled cRNA was carried out using the ENZO RNA transcript labeling kit (Affymetrix). For hybridization, 15 μg fragmented cRNA were incubated with the chip in 200 μl hybridization solution in Hybridization Oven 640 (Affymetrix) at 45°C for 16 hours. Genechips were washed and stained with streptavidin-phycoerythrin using the microfluidic workstation™ (Affymetrix) and scanned with a laser scanner (Agilent Technologies, Böblingen, Germany).

### Microarray quantification and statistical analysis

Quality controls were performed using Affymetrix Microarray Suite 5.0 (MAS 5.0) software. Data and statistical analysis and data visualization were performed with GeneSpring™ (Agilent Technologies). Expression values were extracted from the CEL file, and imported to GeneSpring using robust multi-array average for further analysis [26]. Two criteria were used to select for significant changes in gene expression. First, probe sets with less than 1.2-fold change on comparing *Nfia*^-/- ^with *Nfia*^+/+ ^(control) samples were removed in order to reduce the number of genes undergoing statistical analysis. Second, genes were then tested statistically for significant changes in all three wild-type or NFI-A deficient samples, using Student's *t*-test and multiple corrections with a false discovery rate of 5%. Finally, genes were grouped based on their biologic function using Affymetrix NetAffx and the National Center for Biotechnology Information's Locuslink.

**Figure 9:**
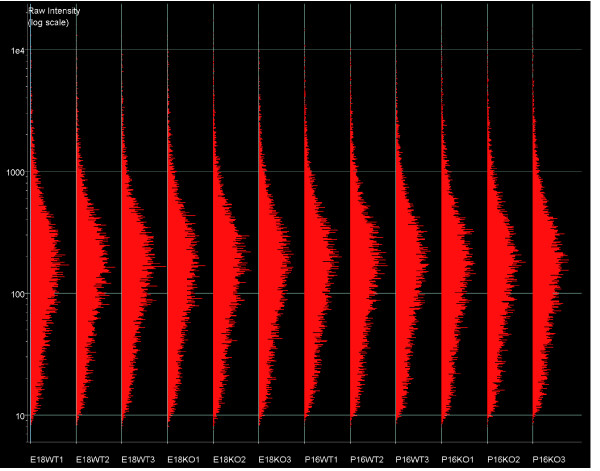
Distribution of raw signal intensities on all microarrays used (non-normalized). E, embryonic day; KO, knockout (*Nfia*^-/-^); P, postnatal day; WT, wild-type (*Nfia*^+/+^).

**Figure 10:**
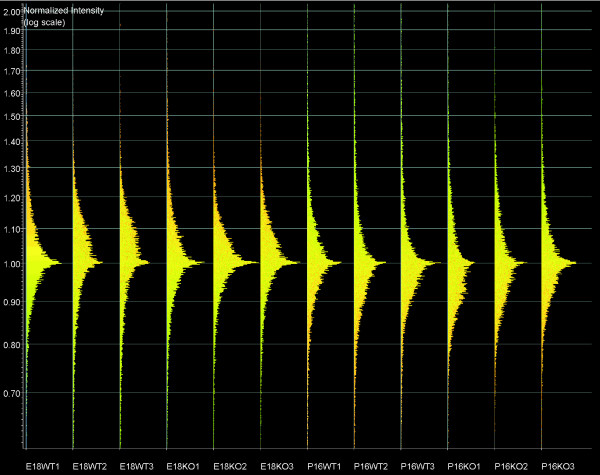
Distribution of normalized signal intensities on all microarrays used (per chip normalization to 50th percentile). Highly similar intensity patterns were observed for all chips used in this study, confirming that all microarray chips in these experiments are comparable. E, embryonic day; KO, knockout (*Nfia*^-/-^); P, postnatal day; WT, wild-type (*Nfia*^+/+^).

All microarrays used in this study exhibit a comparable distribution of signal intensities (Figures 9 and 10), fulfilling the requirements for performing gene expression comparisons. Microarray quality control parameters are summarized in Additional data file 4.

### Accession numbers

Related microarray data are deposited at Gene Expression Omnibus under the following GenBank accession numbers: GSM103419, GSM103420, GSM103421, GSM103422, GSM103423, GSM103424, GSM103425, GSM103426, GSM103427, GSM103428, GSM103429, and GSM103430.

### Reverse transcription and quantitative real-time PCR

The reverse transcription was performed using Superscript II (Invitrogen), in accordance with the manufacturer's protocol. Briefly, 5 μg total RNA was reverse transcribed with random hexamers. First-strand reverse transcribed cDNA was then diluted 1:10 in water before use for real-time PCR. Primers (Metabion, Martinsried, Germany), as listed in Table 6, were used together with the qPCR Core Kit (Eurogentec, Köln, Germany) in an ABI 7900 HT system (Applied Biosystems, Darmstadt, Germany), in accordance with the manufacturer's instructions. Real-time PCR data analysis was performed using the comparative C_T _method (Applied Biosystems), with hypoxanthine guanine phosphoribosyl transferase (Hprt) as an endogenous reference. Statistical significance of expression differences detected by qRT-PCR was examined using a two-tailed, two-sample equal variance *t*-test.

**Table 6:** Primer sequences used for real-time PCR

Gene	Forward primer	Reverse primer
*Car2*	CTGACCACTCCGCCTCTG	AGCGTACGGAAATGAGACATC
*Dcx*	ACACCCTTGATGGAAAGCAG	AGGACCACAAGCAATGAACA
*DIO2*	GTAGCCTTTGAACGTGTGTGCA	TTCTCCAGCCAACTTCGGACT
*Gabra6*	TGGAAGCGGAGATTGTTGTG	CAGGCGTCGATTTTAAGATGG
*Gfap*	GCTGGAGGGCGAAGAAAAC	GGCCTTCTGACACGGATTTG
*Hprt*	GTTCTTTGCTGACCTGCTGGA	TCCCCCGTTGACTGATCATT
*Mag*	TCTCTACCCGGGATTGTCAC	AGGTCCAGTTCTGGGGATTC
*Mal*	CCTCAGTGCCTCAGTTCTGG	GTGACCACGTAGGCAAACAC
*Mobp*	CACGGATGAAAACCCAGTGAG	TCACGCTTGGAGTTGAGGAAG
*Mog*	GAATCTCCATCGGACTTTTGA	GGTCCAAGAACAGGCACAAT
*Nnat*	AGTAGACCTCGGCGAACCCT	CCCAGTAAATGCAGCATTCCA
*Plp1*	TGCGCTGATGCCAGAATGTA	GCGAAGTTGTAAGTGGCAGCA
*Sox2*	GCAGTTAAATTTAGGACCGTTACAA	TCTCATGTTTTCCTTTTGTACAATTT
*Sox4*	GGACAGCGACAAGATTCCGTT	TGCCCGACTTCACCTTCTTTC
*Sox11*	CAAGGTATTCCAGCTACTGGCC	CGGCTAGACTGCTATGCACACA
*TN-C*	GGCGTTAACTGGTTCCATTGG	ATTTATGCCCGCTTACGCCTG

### Computer-based promoter analysis

For each given Affymetrix probe set identity (ID), the corresponding Ensembl gene IDs were retrieved from Ensembl [44]. Promoter sequences were then downloaded from Ensembl (release 34, October 2005). One species was defined as reference species (*Mus musculus*), and orthologous genes from *Homo sapiens *and *Rattus norvegicus *were extracted. For each gene, a sequence corresponding to 2 kb upstream of the known transcription start site of each transcript was taken. In case of genes yielding more than one transcript, the window was enlarged to give a stretch of sequence ranging from 2 kb upstream the most 5' starting transcript to the 5' end of the most downstream starting transcript. No repeat masking was involved. Each set of orthologous sequences was screened for the presence of NFI motifs given a threshold for the score of 80%. The NFI motif is represented by a positional weight matrix. All possible permutations of detected sites from all species under study were aligned using ClustalW. The 'percentage identity' value, as calculated by ClustalW, was taken as motif conservation. Sets with a conservation better than 80% were reported.

In total, 395 Affymetrix probe sets resulted in 714 Ensembl gene IDs. The motif finding process detected 172 conserved motifs corresponding to 83 Affymetrix probe names, 88 Ensembl gene IDs, 79 unique gene symbols, and 128 motifs (Additional data file 3).

## Additional data files

The following additional data are available with the online version of this paper. Additional data file 1 provides a table listing genes significantly dysregulated in *Nfia*^-/- ^mice at E18 or P16, according to microarray analysis. Additional data file 2 provides a table displaying the functional assignment of genes dysregulated in P16 *Nfia*^-/- ^mice. Additional data file 3 provides a table listing putative NFI binding sites in the promoters of genes dysregulated in P16 *Nfia*^-/- ^mice. Additional data file 4 provides a table summarizing microarray quality control parameters.

## Supplementary Material

Additional data file 1 Provided is a table listing genes significantly dysregulated in *Nfia*^-/- ^mice at E18 or P16, according to microarray analysis. Click here for file

Additional data file 2 Provided is a table displaying the functional assignment of genes dysregulated in P16 *Nfia*^-/- ^mice. Click here for file

Additional data file 3 Provided is a table listing putative NFI binding sites in the promoters of genes dysregulated in P16 *Nfia*^-/- ^mice. Click here for file

Additional data file 4 Provided is a table summarizing microarray quality control parameters. Click here for file
