# Supplementary material for: Gene expression analysis of nuclear factor I-A deficient mice indicates delayed brain maturation
Source: Genome Biol. 2007 May 2;8(5):R72. doi: 10.1186/gb-2007-8-5-r72 (PMC1929142; doi:10.1186/gb-2007-8-5-r72)
Supplement: Additional data file 1 — Provided is a table listing genes significantly dysregulated in Nfia-/- mice at E18 or P16, according to microarray analysis. [file gb-2007-8-5-r72-S1.doc]

**Additional data file 1 (Wong et al.)**

**Genes significantly dysregulated in *Nfia^-/-^*** mice at embryonic day 18 according to microarray analysis

| **Gene Symbol** | **Name of gene product** | **Affymetrix probe set ID** | **fold change^1^** | **p value^2^** |
| --- | --- | --- | --- | --- |
| Pip5k2c | phosphatidylinositol-4-phosphate 5-kinase, type II, gamma | 161763_r_at | -1.40 | 0.0169 |
| 1110057K04Rik | RIKEN cDNA 1110057K04 gene | 161190_r_at | -1.30 | 0.0040 |
| Syncrip | Synaptotagmin binding, cytoplasmic RNA interacting protein | 96375_at | -1.23 | 0.0407 |
| Plagl1 | pleiomorphic adenoma gene-like 1 | 92502_at | 1.23 | 0.0230 |
| Syt1 | synaptotagmin 1 | 93005_at | 1.29 | 0.0063 |

**^1^** in *Nfia^-/-^* mice relative to *Nfia^+/+^* mice. Only probe sets with fold changes of more than 1.20 were considered as representing significantly dysregulated transcripts.

**^2^** only probe sets with p values < 0.05 were considered as representing significantly dysregulated transcripts

**Genes significantly dysregulated in *Nfia^-/-^* mice at postnatal day 16 according to microarray analysis**

Genes represented by more than one probe set are labeled in blue, probe sets probably representing more than one gene are labeled in red.

| **Gene Symbol** | **Name of gene product^3^** | **Affymetrix probe set ID** | **fold change^1^** | **p value^2^** |
| --- | --- | --- | --- | --- |
| Gabra6 | gamma-aminobutyric acid (GABA-A) receptor, subunit alpha 6 | 92939_at | -3.22 | 0.0026 |
| Aldh1a1 | aldehyde dehydrogenase family 1, subfamily A1 | 100068_at | -3.14 | 0.0075 |
| Gabra6 | gamma-aminobutyric acid (GABA-A) receptor, subunit alpha 6 | 92940_s_at | -2.94 | 0.0018 |
| Mal | myelin and lymphocyte protein, T-cell differentiation protein | 99089_at | -2.43 | 0.0007 |
| Agt | angiotensinogen | 101887_at | -2.24 | 0.0007 |
| Ntsr2 | neurotensin receptor 2 | 93137_at | -2.21 | 0.0003 |
| Lcat | lecithin cholesterol acyltransferase | 103023_at | -2.13 | 0.0003 |
| Mobp | myelin-associated oligodendrocytic basic protein | 99046_at | -2.11 | 0.0080 |
| Mobp | myelin-associated oligodendrocytic basic protein | 100536_at | -2.10 | 0.0048 |
| Gjb6 | gap junction membrane channel protein beta 6 | 94391_at | -2.07 | 0.0014 |
| Folh1 | folate hydrolase | 97089_at | -2.06 | 0.0128 |
| Mobp | myelin-associated oligodendrocytic basic protein | 99047_at | -1.99 | 0.0011 |
| Mobp | myelin-associated oligodendrocytic basic protein | 99048_g_at | -1.98 | 0.0022 |
| Pygm | muscle glycogen phosphorylase | 160754_at | -1.97 | 0.0059 |
| Mog | myelin oligodendrocyte glycoprotein | 103987_at | -1.95 | 0.0044 |
| S100b | S100 protein, beta polypeptide, neural | 101467_at | -1.90 | 0.0018 |
| Itih3 | inter-alpha trypsin inhibitor, heavy chain 3 | 100002_at | -1.89 | 0.0016 |
| Clu | clusterin | 95286_at | -1.88 | 0.0112 |
| Fgf1 | Fibroblast growth factor 1 | 100494_at | -1.87 | 0.0003 |
| Gjb6 | gap junction membrane channel protein beta 6 | 102571_at | -1.81 | 0.0194 |
| Enpp2 | ectonucleotide pyrophosphatase/phosphodiesterase 2 | 97317_at | -1.81 | 0.0003 |
| Gfap | Glial fibrillary acidic protein | 94144_g_at | -1.71 | 0.0053 |
| Csrp1 | cysteine and glycine-rich protein 1 | 160065_s_at | -1.70 | 0.0066 |
| Scd1 | stearoyl-Coenzyme A desaturase 1 | 94057_g_at | -1.70 | 0.0227 |
| Ndrg2 | N-myc downstream regulated gene 2 | 161610_at | -1.69 | 0.0053 |
| Ndrg2 | N-myc downstream regulated gene 2 | 96088_at | -1.68 | 0.0021 |
| Neurod1 | Neurogenic differentiation 1 | 92717_at | -1.68 | 0.0135 |
| Sept4 | septin 4 | 94079_at | -1.68 | 0.0035 |
| Scd1 | stearoyl-Coenzyme A desaturase 1 | 94056_at | -1.68 | 0.0093 |
| Prom1 | prominin 1 | 93389_at | -1.67 | 0.0026 |
| Ugt8 | UDP-glucuronosyltransferase 8 | 98872_at | -1.67 | 0.0129 |
| Mt1 | metallothionein 1 | 93573_at | -1.67 | 0.0396 |
| Prom1 | prominin 1 | 93390_g_at | -1.67 | 0.0043 |
| Pvalb | parvalbumin | 96720_f_at | -1.66 | 0.0145 |
| Ank1 | ankyrin 1, erythroid | 100441_s_at | -1.64 | 0.0014 |
| Csrp1 | cysteine and glycine-rich protein 1 | 92608_at | -1.63 | 0.0194 |
| Clu | clusterin | 161294_f_at | -1.63 | 0.0093 |
| AL024210 | Expressed sequence AL024210 | 103429_i_at | -1.61 | 0.0060 |
| Cldn11 | claudin 11 | 100044_at | -1.58 | 0.0196 |
| Gsn | gelsolin | 93750_at | -1.58 | 0.0011 |
| Gfap | Glial fibrillary acidic protein | 94143_at | -1.56 | 0.0279 |
| Plp1 | Proteolipid protein (myelin) 1 | 92802_s_at | -1.54 | 0.0333 |
| R74640 | Expressed sequence R74640 | 93159_at | -1.54 | 0.0065 |
| Cbln1 | Cerebellin 1 precursor protein | 92932_at | -1.52 | 0.0059 |
| Mag | Myelin-associated glycoprotein | 102405_at | -1.52 | 0.0111 |
| En2 | engrailed 2 | 98338_at | -1.52 | 0.0363 |
| Atp1b2 | ATPase, Na+/K+ transporting, beta 2 polypeptide | 93664_at | -1.51 | 0.0272 |
| Evi2a | Ecotropic viral integration site 2a | 98025_at | -1.51 | 0.0147 |
| Car8 | carbonic anhydrase 8 | 102773_at | -1.50 | 0.0246 |
| Car4 | carbonic anhydrase 4 | 103046_at | -1.50 | 0.0079 |
| Mt2 | metallothionein 2 | 101561_at | -1.49 | 0.0080 |
| Myoc | myocilin | 94122_at | -1.49 | 0.0119 |
| Ttr | transthyretin | 95350_at | -1.48 | 0.0248 |
| Cst3 | cystatin C | 99586_at | -1.47 | 0.0001 |
| Thrsp | Thyroid hormone responsive SPOT14 homolog (Rattus) | 160306_at | -1.47 | 0.0170 |
| Scn1b | sodium channel, voltage-gated, type I, beta polypeptide | 102808_at | -1.46 | 0.0191 |
| Gpr37l1 | G protein-coupled receptor 37-like 1 | 102305_at | -1.46 | 0.0001 |
| Cst3 | cystatin C | 161522_i_at | -1.46 | 0.0055 |
| Atp1a2 | ATPase, Na+/K+ transporting, alpha 2 polypeptide | 99481_at | -1.45 | 0.0001 |
| Plp1 | Proteolipid protein (myelin) 1 | 92801_at | -1.45 | 0.0051 |
| Nefh | Neurofilament, heavy polypeptide | 103234_at | -1.45 | 0.0011 |
| Il16 | interleukin 16 | 102029_at | -1.44 | 0.0129 |
| Dio2 | Deiodinase, iodothyronine, type II | 103438_at | -1.43 | 0.0072 |
| Scrg1 | Scrapie responsive gene 1 | 94181_at | -1.43 | 0.0083 |
| Btbd3 | BTB (POZ) domain containing 3 | 95393_at | -1.43 | 0.0013 |
| Aldoc | aldolase 3, C isoform | 160546_at | -1.43 | 0.0002 |
| Pla2g7 | Phospholipase A2, group VII (platelet-activating factor acetylhydrolase, plasma) | 101923_at | -1.41 | 0.0055 |
| Selpl | selectin, platelet (p-selectin) ligand | 103488_at | -1.41 | 0.0049 |
| Itpr1 | inositol 1,4,5-triphosphate receptor 1 | 93895_s_at | -1.41 | 0.0034 |
| Itpr1 | inositol 1,4,5-triphosphate receptor 1 | 94977_at | -1.41 | 0.0080 |
| Ank1 | ankyrin 1, erythroid | 100440_f_at | -1.40 | 0.0317 |
| Lats2 | Large tumor suppressor 2 | 160261_i_at | -1.40 | 0.0203 |
| Erbb3 | v-erb-b2 erythroblastic leukemia viral oncogene homolog 3 (avian) | 96771_at | -1.40 | 0.0312 |
| Scn1a | sodium channel, voltage-gated, type I, alpha | 94201_at | -1.39 | 0.0358 |
| Prkcd | protein kinase C, delta | 104531_at | -1.38 | 0.0054 |
| Penk1 | preproenkephalin 1 | 94516_f_at | -1.38 | 0.0265 |
| Adssl1 | Adenylosuccinate synthetase like 1 | 98435_at | -1.37 | 0.0062 |
| Mpp3 | Membrane protein, palmitoylated 3 (MAGUK p55 subfamily member 3) | 103247_at | -1.37 | 0.0109 |
| Gamt | Guanidinoacetate methyltransferase | 101408_at | -1.37 | 0.0100 |
| Rnf13 | ring finger protein 13 | 101966_s_at | -1.37 | 0.0234 |
| Hist1h2bc | histone 1, H2bc | 93833_s_at | -1.37 | 0.0129 |
| Prss11 | Protease, serine, 11 (Igf binding) | 96920_at | -1.36 | 0.0006 |
| Fnbp1 | Formin binding protein 1 | 103638_at | -1.36 | 0.0050 |
| Piga | Phosphatidylinositol glycan, class A | 161433_f_at | -1.36 | 0.0021 |
| 1110011C06Rik | RIKEN cDNA 1110011C06 gene | 98464_at | -1.35 | 0.0124 |
| Efhd1 | EF hand domain containing 1 | 92786_at | -1.35 | 0.0124 |
| Hr | hairless | 99875_at | -1.35 | 0.0126 |
| Bcan | brevican | 92700_at | -1.35 | 0.0064 |
| Anln | anillin, actin binding protein (scraps homolog, Drosophila) | 96784_at | -1.35 | 0.0053 |
| Car2 | carbonic anhydrase 2 | 92642_at | -1.35 | 0.0057 |
| Sdc4 | syndecan 4 | 98590_at | -1.34 | 0.0210 |
| Vamp1 | Vesicle-associated membrane protein 1 | 93652_i_at | -1.34 | 0.0180 |
| Edg2 | Endothelial differentiation, lysophosphatidic acid G-protein-coupled receptor, 2 | 100435_at | -1.34 | 0.0082 |
| Eno2 | Enolase 2, gamma neuronal | 99045_at | -1.34 | 0.0374 |
| Eps8 | Epidermal growth factor receptor pathway substrate 8 | 103222_at | -1.34 | 0.0394 |
| Mcam | Melanoma cell adhesion molecule | 160458_at | -1.33 | 0.0100 |
| Apod | apolipoprotein D | 93592_at | -1.33 | 0.0389 |
| Tmeff2 | Transmembrane protein with EGF-like and two follistatin-like domains 2 | 103536_at | -1.33 | 0.0104 |
| Abcb1a | ATP-binding cassette, sub-family B (MDR/TAP), member 1A | 102910_at | -1.32 | 0.0224 |
| 1110007C05Rik | RIKEN cDNA 1110007C05 gene | 95041_at | -1.32 | 0.0134 |
| Apoe | apolipoprotein E | 95356_at | -1.31 | 0.0005 |
| Rora | RAR-related orphan receptor alpha | 101889_s_at | -1.30 | 0.0092 |
| Gpr83 | G protein-coupled receptor 83 | 92734_at | -1.30 | 0.0286 |
| 2900060B22Rik | RIKEN cDNA 2900060B22 gene | 96590_f_at | -1.29 | 0.0435 |
| Prkcd | protein kinase C, delta | 160698_s_at | -1.29 | 0.0192 |
| Tubb4 | tubulin, beta 4 | 101419_at | -1.29 | 0.0444 |
| 2900092E17Rik | RIKEN cDNA 2900092E17 gene | 95759_at | -1.29 | 0.0009 |
| Gna12 | guanine nucleotide binding protein, alpha 12 | 97227_at | -1.29 | 0.0231 |
| Kcnk1 | potassium channel, subfamily K, member 1 | 102335_at | -1.29 | 0.0333 |
| 6530413N01Rik | RIKEN cDNA 6530413N01 gene | 99553_f_at | -1.28 | 0.0088 |
| Gabrd | gamma-aminobutyric acid (GABA-A) receptor, subunit delta | 99342_at | -1.28 | 0.0117 |
| 0610009M14Rik | RIKEN cDNA 0610009M14 gene | 94229_at | -1.28 | 0.0001 |
| Calb2 | calbindin 2 | 95036_at | -1.28 | 0.0029 |
| 4631408O11Rik | RIKEN cDNA 4631408O11 gene | 104445_at | -1.28 | 0.0086 |
| Hsd11b1 | hydroxysteroid 11-beta dehydrogenase 1 | 97867_at | -1.27 | 0.0219 |
| Pvalb | parvalbumin | 96719_i_at | -1.27 | 0.0076 |
| Epas1 | endothelial PAS domain protein 1 | 102698_at | -1.27 | 0.0369 |
| Pcaf | p300/CBP-associated factor | 104070_at | -1.27 | 0.0310 |
| Kcnab2 | potassium voltage-gated channel, shaker-related subfamily, beta member 2 | 102892_at | -1.27 | 0.0089 |
| NF-L | Mouse neurofilament protein (NF-L) gene, 3' flank | 103575_at | -1.27 | 0.0055 |
| Sncg | synuclein, gamma | 104280_at | -1.27 | 0.0087 |
| Snrk | SNF related kinase | 97429_at | -1.27 | 0.0247 |
| Entpd2 | ectonucleoside triphosphate diphosphohydrolase 2 | 97986_at | -1.27 | 0.0281 |
| D2Bwg0891e | DNA segment, Chr 2, Brigham & Women's Genetics 0891 expressed | 99641_at | -1.26 | 0.0327 |
| Ndrg1 /// Ndrl | N-myc downstream regulated gene 1 /// N-myc downstream regulated-like | 96596_at | -1.26 | 0.0007 |
| Nr1d2 | nuclear receptor subfamily 1, group D, member 2 | 99076_at | -1.26 | 0.0169 |
| Pacsin1 | protein kinase C and casein kinase substrate in neurons 1 | 92949_at | -1.26 | 0.0078 |
| Rps6ka1 | ribosomal protein S6 kinase polypeptide 1 | 97405_at | -1.26 | 0.0294 |
| Gpd1 | glycerol-3-phosphate dehydrogenase 1 (soluble) | 92592_at | -1.26 | 0.0064 |
| Scn1a | sodium channel, voltage-gated, type I, alpha | 162255_s_at | -1.26 | 0.0372 |
| Cds2 | CDP-diacylglycerol synthase (phosphatidate cytidylyltransferase) 2 | 161773_i_at | -1.26 | 0.0486 |
| Sdha | succinate dehydrogenase complex, subunit A, flavoprotein (Fp) | 94080_at | -1.25 | 0.0044 |
| Cav1 | caveolin, caveolae protein 1 | 160280_at | -1.25 | 0.0013 |
| Dao1 | D-amino acid oxidase | 103602_at | -1.25 | 0.0272 |
| Nos1 | nitric oxide synthase 1, neuronal | 98365_at | -1.25 | 0.0127 |
| Clic4 | chloride intracellular channel 4 (mitochondrial) | 94256_at | -1.25 | 0.0157 |
| Nef3 | neurofilament 3, medium | 92346_at | -1.25 | 0.0388 |
| Phka1 | phosphorylase kinase alpha 1 | 92519_at | -1.25 | 0.0251 |
| Slc12a2 | solute carrier family 12, member 2 | 99500_at | -1.25 | 0.0137 |
| Grina | glutamate receptor, ionotropic, N-methyl D-asparate-associated protein 1 (glutamate binding) | 99160_s_at | -1.25 | 0.0313 |
| Pip5k1a | phosphatidylinositol-4-phosphate 5-kinase, type 1 alpha | 103573_at | -1.25 | 0.0420 |
| Abca2 | ATP-binding cassette, sub-family A (ABC1), member 2 | 104137_at | -1.25 | 0.0296 |
| Phldb1 | pleckstrin homology-like domain, family B, member 1 | 96206_at | -1.25 | 0.0004 |
| 2810012G08Rik | RIKEN cDNA 2810012G08 gene | 102942_at | -1.24 | 0.0213 |
| Phkg1 | phosphorylase kinase gamma 1 | 97094_at | -1.24 | 0.0067 |
| Ldlr | Low density lipoprotein receptor | 160832_at | -1.24 | 0.0080 |
| Sparcl1 | SPARC-like 1 (mast9, hevin) | 160319_at | -1.24 | 0.0121 |
| Hadhb | hydroxyacyl-Coenzyme A dehydrogenase/3-ketoacyl-Coenzyme A thiolase/enoyl-Coenzyme A hydratase (trifunctional protein), beta subunit | 96913_at | -1.23 | 0.0066 |
| Cplx1 | complexin 1 | 101198_at | -1.23 | 0.0043 |
| Nkx6-2 | NK6 transcription factor related, locus 2 (Drosophila) | 93182_at | -1.23 | 0.0026 |
| Fnbp1 | Formin binding protein 1 | 98729_at | -1.23 | 0.0080 |
| Rgs16 | regulator of G-protein signaling 16 | 161609_at | -1.23 | 0.0005 |
| C130065N10Rik | RIKEN cDNA C130065N10 gene | 92408_at | -1.23 | 0.0048 |
| 4930422J18Rik | RIKEN cDNA 4930422J18 gene | 161013_f_at | -1.22 | 0.0363 |
| Padi2 | peptidyl arginine deiminase, type II | 103526_at | -1.22 | 0.0254 |
| Zfp179 | zinc finger protein 179 | 100383_at | -1.22 | 0.0217 |
| Adarb1 | adenosine deaminase, RNA-specific, B1 | 161436_s_at | -1.22 | 0.0338 |
| Flt1 | FMS-like tyrosine kinase 1 | 98452_at | -1.22 | 0.0014 |
| Myl4 | myosin, light polypeptide 4 | 160487_at | -1.22 | 0.0409 |
| Ak3l1 | adenylate kinase 3 alpha-like 1 | 99959_at | -1.22 | 0.0288 |
| Kcnk3 | potassium channel, subfamily K, member 3 | 102020_at | -1.22 | 0.0393 |
| C330016K18Rik | RIKEN cDNA C330016K18 gene | 96599_at | -1.22 | 0.0450 |
| Tyro3 | TYRO3 protein tyrosine kinase 3 | 96766_s_at | -1.22 | 0.0162 |
| MGI:1333876 | G substrate | 98293_g_at | -1.21 | 0.0160 |
| Kcnab1 | potassium voltage-gated channel, shaker-related subfamily, beta member 1 | 102725_at | -1.21 | 0.0203 |
| Dusp16 | dual specificity phosphatase 16 | 97740_at | -1.21 | 0.0440 |
| Qdpr | quininoid dihydropteridine reductase | 96948_at | -1.21 | 0.0007 |
| 1190002N15Rik | RIKEN cDNA 1190002N15 gene | 98594_at | -1.21 | 0.0264 |
| Eln | elastin | 92836_at | -1.21 | 0.0157 |
| Shox2 | short stature homeobox 2 | 99042_s_at | -1.21 | 0.0215 |
| B830009D23Rik | RIKEN cDNA B830009D23 gene | 93179_at | -1.21 | 0.0263 |
| Phlda3 | pleckstrin homology-like domain, family A, member 3 | 98056_at | -1.21 | 0.0203 |
| Gm98 | gene model 98, (NCBI) | 104266_at | -1.21 | 0.0013 |
| AA960558 | expressed sequence AA960558 | 94359_at | -1.21 | 0.0286 |
| Adk | adenosine kinase | 93512_f_at | -1.20 | 0.0393 |
| Ctss | cathepsin S | 98543_at | -1.20 | 0.0288 |
| 4933426M11Rik | RIKEN cDNA 4933426M11 gene | 96614_at | -1.20 | 0.0379 |
| Tbc1d15 | TBC1 domain family, member 15 | 103471_at | -1.20 | 0.0151 |
| Apaf1 | apoptotic protease activating factor 1 | 103796_at | 1.20 | 0.0427 |
| Dbi | diazepam binding inhibitor | 97248_at | 1.20 | 0.0193 |
| Rbbp7 | retinoblastoma binding protein 7 | 93081_at | 1.20 | 0.0320 |
| Lars | leucyl-tRNA synthetase | 103630_at | 1.20 | 0.0350 |
| 2310008M10Rik | RIKEN cDNA 2310008M10 gene | 160569_at | 1.20 | 0.0014 |
| Cct4 | chaperonin subunit 4 (delta) | 101011_at | 1.20 | 0.0326 |
| Ly6h | lymphocyte antigen 6 complex, locus H | 103487_at | 1.20 | 0.0161 |
| Myef2 | myelin basic protein expression factor 2, repressor | 160626_at | 1.20 | 0.0036 |
| Rpl19 | ribosomal protein L19 | 97483_at | 1.20 | 0.0304 |
| Rpl8 | ribosomal protein L8 | 96575_at | 1.20 | 0.0309 |
| Lsm3 | LSM3 homolog, U6 small nuclear RNA associated (S. cerevisiae) | 94455_at | 1.20 | 0.0244 |
| Myo1b | myosin IB | 98409_at | 1.20 | 0.0380 |
| Rbm8a | RNA binding motif protein 8a | 97254_at | 1.20 | 0.0459 |
| Aurka | aurora kinase A | 92639_at | 1.20 | 0.0288 |
| Odc1 | ornithine decarboxylase, structural 1 | 160084_at | 1.21 | 0.0012 |
| Pkia | protein kinase inhibitor, alpha | 98004_at | 1.21 | 0.0129 |
| Ptgds | prostaglandin D2 synthase (brain) | 102105_f_at | 1.21 | 0.0402 |
| Magoh | mago-nashi homolog, proliferation-associated (Drosophila) | 160550_i_at | 1.21 | 0.0364 |
| Rps28 | B6-derived CD11 +ve dendritic cells cDNA, RIKEN full-length enriched library, clone:F730014O09 product:40S RIBOSOMAL PROTEIN S28 homolog [Ictalurus punctatus], full insert sequence | 98085_f_at | 1.21 | 0.0163 |
| Suclg2 | succinate-Coenzyme A ligase, GDP-forming, beta subunit | 160428_at | 1.21 | 0.0042 |
| Tuba1 | tubulin, alpha 1 | 100343_f_at | 1.21 | 0.0030 |
| 2700069A02Rik | RIKEN cDNA 2700069A02 gene | 97380_at | 1.21 | 0.0309 |
| Hmgb1 | high mobility group box 1 | 93095_at | 1.21 | 0.0166 |
| Stmn2 | stathmin-like 2 | 95669_g_at | 1.21 | 0.0049 |
| Cdca8 | cell division cycle associated 8 | 97295_at | 1.21 | 0.0020 |
| Nf2 | neurofibromatosis 2 | 92643_at | 1.21 | 0.0153 |
| Lsm7 | LSM7 homolog, U6 small nuclear RNA associated (S. cerevisiae) | 97907_at | 1.21 | 0.0370 |
| Igfbpl1 | insulin-like growth factor binding protein-like 1 | 92349_at | 1.21 | 0.0143 |
| Lsm4 | LSM4 homolog, U6 small nuclear RNA associated (S. cerevisiae) | 93008_at | 1.21 | 0.0254 |
| Trim28 | tripartite motif protein 28 | 93071_at | 1.21 | 0.0005 |
| Rpl23a /// LOC270584 /// LOC383850 | ribosomal protein L23a /// similar to 60S ribosomal protein L23a /// similar to 60S ribosomal protein L23a | 94823_at | 1.21 | 0.0396 |
| Rpl36a | ribosomal protein L36a | 160081_at | 1.21 | 0.0314 |
| Rps3 | ribosomal protein S3 | 101137_at | 1.21 | 0.0047 |
| Sept9 | septin 9 | 98609_at | 1.21 | 0.0054 |
| Hnrpa2b1 | heterogeneous nuclear ribonucleoprotein A2/B1 | 93117_at | 1.22 | 0.0230 |
| Polr2d | polymerase (RNA) II (DNA directed) polypeptide D | 97315_at | 1.22 | 0.0114 |
| Gnb4 | guanine nucleotide binding protein, beta 4 | 93949_at | 1.22 | 0.0084 |
| Mrpl52 | mitochondrial ribosomal protein L52 | 97443_at | 1.22 | 0.0487 |
| Rpl3 | ribosomal protein L3 | 100734_at | 1.22 | 0.0089 |
| Rplp1 | ribosomal protein, large, P1 | 100694_at | 1.22 | 0.0238 |
| Adh5 | alcohol dehydrogenase 5 (class III), chi polypeptide | 98625_s_at | 1.22 | 0.0056 |
| Rpl22 | ribosomal protein L22 | 92857_at | 1.22 | 0.0296 |
| 2700094K13Rik | RIKEN cDNA 2700094K13 gene | 96016_at | 1.22 | 0.0036 |
| 2900010J23Rik | RIKEN cDNA 2900010J23 gene | 96658_at | 1.22 | 0.0361 |
| Lpl | lipoprotein lipase | 160083_at | 1.22 | 0.0076 |
| Mapk8 | mitogen activated protein kinase 8 | 104047_at | 1.22 | 0.0018 |
| Rps27 | ribosomal protein S27 | 96300_f_at | 1.23 | 0.0038 |
| Rpl18 | ribosomal protein L18 | 160476_f_at | 1.23 | 0.0172 |
| Ranbp1 | RAN binding protein 1 | 98573_r_at | 1.23 | 0.0254 |
| Rpl36 | ribosomal protein L36 | 92628_at | 1.23 | 0.0174 |
| Ranbp5 | RAN binding protein 5 | 93070_at | 1.23 | 0.0180 |
| Evl | Ena-vasodilator stimulated phosphoprotein | 160667_at | 1.23 | 0.0160 |
| Sin3b | transcriptional regulator, SIN3B (yeast) | 93789_s_at | 1.23 | 0.0056 |
| Sfpq | splicing factor proline/glutamine rich (polypyrimidine tract binding protein associated) | 99620_at | 1.23 | 0.0490 |
| Agrn | agrin | 97921_at | 1.23 | 0.0265 |
| Gng2 | guanine nucleotide binding protein (G protein), gamma 2 subunit | 100418_at | 1.23 | 0.0053 |
| Pcdha11 | protocadherin alpha 11 | 101650_at | 1.23 | 0.0047 |
| Pkia | protein kinase inhibitor, alpha | 98005_at | 1.23 | 0.0057 |
| Anapc5 | anaphase-promoting complex subunit 5 | 95100_at | 1.23 | 0.0018 |
| Rpl7 | ribosomal protein L7 | 97696_r_at | 1.24 | 0.0050 |
| Basp1 | Brain abundant, membrane attached signal protein 1 | 95673_s_at | 1.24 | 0.0033 |
| Rbmxrt | RNA binding motif protein, X chromosome retrogene | 160192_at | 1.24 | 0.0151 |
| Rps16 | ribosomal protein S16 | 97647_at | 1.24 | 0.0278 |
| Epha5 | Eph receptor A5 | 161119_at | 1.24 | 0.0083 |
| Rps27a | ribosomal protein S27a | 93030_at | 1.24 | 0.0050 |
| Zfp90 | zinc finger protein 90 | 92934_at | 1.24 | 0.0008 |
| Rps2 | ribosomal protein S2 | 100686_at | 1.24 | 0.0009 |
| Rps11 | Ribosomal protein S11 | 94767_at | 1.24 | 0.0302 |
| Tubb2 | tubulin, beta 2 | 94835_f_at | 1.24 | 0.0075 |
| Rpl7a | Ribosomal protein L7a | 98168_at | 1.25 | 0.0406 |
| Vcam1 | vascular cell adhesion molecule 1 | 92558_at | 1.25 | 0.0064 |
| 1100001I22Rik /// LOC384425 /// MGC103270 | RIKEN cDNA 1100001I22 gene /// similar to 60S ribosomal protein L34 /// ribosomal protein L34 | 96307_s_at | 1.25 | 0.0072 |
| Deaf1 | deformed epidermal autoregulatory factor 1 (Drosophila) | 96171_at | 1.25 | 0.0489 |
| Rbmx | RNA binding motif protein, X chromosome | 97847_at | 1.25 | 0.0284 |
| Pafah1b3 | Platelet-activating factor acetylhydrolase, isoform 1b, alpha1 subunit | 100576_at | 1.25 | 0.0426 |
| Tuba1 | tubulin, alpha 1 | 100342_i_at | 1.25 | 0.0257 |
| Hmgn1 | high mobility group nucleosomal binding domain 1 | 96699_at | 1.25 | 0.0087 |
| Mmp14 | matrix metalloproteinase 14 (membrane-inserted) | 160118_at | 1.25 | 0.0210 |
| Ptgds | Prostaglandin D2 synthase (brain) | 92546_r_at | 1.25 | 0.0135 |
| Cdk4 | Cyclin-dependent kinase 4 | 160538_at | 1.25 | 0.0009 |
| Efnb2 | ephrin B2 | 160857_at | 1.25 | 0.0297 |
| Rps10 | Ribosomal protein S10 | 99093_at | 1.26 | 0.0447 |
| Rpl12 | Ribosomal protein L12 | 99778_at | 1.26 | 0.0292 |
| Rpl26 | Ribosomal protein L26 | 100729_at | 1.26 | 0.0369 |
| Rpl27a /// LOC432798 /// LOC433510 | ribosomal protein L27a /// similar to 60S ribosomal protein L27a (L29) /// similar to ribosomal protein L27a; ribosomal protein L29 homolog (yeast) | 101680_at | 1.26 | 0.0006 |
| Snrpd2 | Small nuclear ribonucleoprotein D2 | 95049_at | 1.26 | 0.0245 |
| Ftl1 /// Ftl2 /// Grik3 /// AI642036 | ferritin light chain 1 /// ferritin light chain 2 /// glutamate receptor, ionotropic, kainate 3 /// expressed sequence AI642036 | 99872_s_at | 1.26 | 0.0042 |
| Sfrs10 | splicing factor, arginine/serine-rich 10 (transformer 2 homolog, Drosophila) | 160364_at | 1.26 | 0.0298 |
| Impdh2 | inosine 5'-phosphate dehydrogenase 2 | 100578_at | 1.26 | 0.0009 |
| Plagl1 | pleiomorphic adenoma gene-like 1 | 92502_at | 1.26 | 0.0092 |
| Snrpe | small nuclear ribonucleoprotein E | 97200_f_at | 1.26 | 0.0113 |
| Top2a | topoisomerase (DNA) II alpha | 99578_at | 1.26 | 0.0495 |
| 2610201A13Rik | RIKEN cDNA 2610201A13 gene | 95927_f_at | 1.26 | 0.0430 |
| Gng10 | guanine nucleotide binding protein (G protein), gamma 10 | 99175_at | 1.26 | 0.0264 |
| Rpl27a | ribosomal protein L27a | 101573_f_at | 1.26 | 0.0070 |
| AB023957 | CDNA sequence AB023957 | 96132_at | 1.26 | 0.0037 |
| Npm1 | nucleophosmin 1 | 101634_at | 1.26 | 0.0093 |
| Tbca | tubulin cofactor a | 93333_at | 1.26 | 0.0126 |
| Mef2c | myocyte enhancer factor 2C | 97357_at | 1.26 | 0.0321 |
| Myo5b | myosin Vb | 92254_at | 1.26 | 0.0068 |
| Sec61g | SEC61, gamma subunit | 92636_f_at | 1.27 | 0.0194 |
| Slc39a8 | solute carrier family 39 (metal ion transporter), member 8 | 97442_at | 1.27 | 0.0027 |
| Nfib | nuclear factor I/B | 160859_s_at | 1.27 | 0.0295 |
| Cdkl2 | cyclin-dependent kinase-like 2 (CDC2-related kinase) | 160623_at | 1.27 | 0.0211 |
| Prdx4 | peroxiredoxin 4 | 93495_at | 1.27 | 0.0095 |
| Rrm2 | ribonucleotide reductase M2 | 102001_at | 1.27 | 0.0271 |
| Slc1a4 | solute carrier family 1 (glutamate/neutral amino acid transporter), member 4 | 100943_at | 1.27 | 0.0293 |
| BC037006 | cDNA sequence BC037006 | 96518_at | 1.27 | 0.0116 |
| Rpo2tc1 | RNA polymerase II transcriptional coactivator | 101980_at | 1.27 | 0.0041 |
| Rps5 | ribosomal protein S5 | 99336_at | 1.27 | 0.0047 |
| Tm4sf6 | transmembrane 4 superfamily member 6 | 92555_at | 1.27 | 0.0061 |
| Rpl37 | ribosomal protein L37 | 92577_f_at | 1.27 | 0.0494 |
| Hsbp1 | heat shock factor binding protein 1 | 93743_at | 1.28 | 0.0165 |
| Tyrobp | TYRO protein tyrosine kinase binding protein | 100397_at | 1.28 | 0.0008 |
| Sfrp1 | secreted frizzled-related sequence protein 1 | 97997_at | 1.28 | 0.0136 |
| Sfpq | splicing factor proline/glutamine rich (polypyrimidine tract binding protein associated) | 99621_s_at | 1.28 | 0.0153 |
| Myb | myeloblastosis oncogene | 92644_s_at | 1.28 | 0.0381 |
| Rpl7 | ribosomal protein L7 | 97695_s_at | 1.28 | 0.0096 |
| Syncrip | synaptotagmin binding, cytoplasmic RNA interacting protein | 94985_at | 1.28 | 0.0459 |
| Sox2 | SRY-box containing gene 2 | 100009_r_at | 1.28 | 0.0438 |
| Fabp5 | fatty acid binding protein 5, epidermal | 160544_at | 1.28 | 0.0031 |
| Maged2 | melanoma antigen, family D, 2 | 94289_r_at | 1.28 | 0.0271 |
| Rps3a | ribosomal protein S3a | 101664_at | 1.28 | 0.0201 |
| Taf9 | TAF9 RNA polymerase II, TATA box binding protein (TBP)-associated factor | 93918_at | 1.28 | 0.0125 |
| Rpl5 | ribosomal protein L5 | 101129_at | 1.29 | 0.0324 |
| Mef2c | myocyte enhancer factor 2C | 104592_i_at | 1.29 | 0.0370 |
| Cnn3 | calponin 3, acidic | 160150_f_at | 1.29 | 0.0172 |
| Ncam1 | Neural cell adhesion molecule | 100153_at | 1.29 | 0.0247 |
| H3f3b | H3 histone, family 3B | 100708_at | 1.29 | 0.0001 |
| Emid2 | EMI domain containing 2 | 97982_at | 1.29 | 0.0246 |
| Rpl30 | ribosomal protein L30 | 98119_at | 1.29 | 0.0212 |
| Rps18 | ribosomal protein S18 | 98333_at | 1.29 | 0.0073 |
| Rps23 | ribosomal protein S23 | 96358_at | 1.29 | 0.0048 |
| Erh | Enhancer of rudimentary homolog (Drosophila) | 94040_at | 1.30 | 0.0219 |
| Prc1 | Protein regulator of cytokinesis 1 | 95032_at | 1.30 | 0.0264 |
| Tubb5 | tubulin, beta 5 | 94789_r_at | 1.30 | 0.0012 |
| Hrmt1l2 | heterogeneous nuclear ribonucleoproteins methyltransferase-like 2 (S. cerevisiae) | 96696_at | 1.30 | 0.0102 |
| Mlp | MARCKS-like protein | 97203_at | 1.30 | 0.0171 |
| Rps7 | ribosomal protein S7 | 101212_at | 1.31 | 0.0034 |
| Smc4l1 | SMC4 structural maintenance of chromosomes 4-like 1 (yeast) | 101906_at | 1.31 | 0.0025 |
| Wdr6 | WD repeat domain 6 | 95643_at | 1.31 | 0.0099 |
| Nme2 | Expressed in non-metastatic cells 2, protein | 92625_at | 1.32 | 0.0040 |
| H2afv | H2A histone family, member V | 96710_at | 1.32 | 0.0140 |
| D430019H16Rik | RIKEN cDNA D430019H16 gene | 95397_at | 1.32 | 0.0020 |
| Gnb2-rs1 | Guanine nucleotide binding protein, beta 2, related sequence 1 | 99340_at | 1.32 | 0.0110 |
| Lpl | lipoprotein lipase | 95611_at | 1.32 | 0.0216 |
| Lyl1 | lymphoblastomic leukemia | 100468_g_at | 1.32 | 0.0250 |
| Snrpg | Small nuclear ribonucleoprotein polypeptide G | 93999_at | 1.32 | 0.0127 |
| Cd200 | Cd200 antigen | 101851_at | 1.32 | 0.0001 |
| Psme1 | Proteasome (prosome, macropain) 28 subunit, alpha | 101510_at | 1.32 | 0.0111 |
| Txn1 | thioredoxin 1 | 92807_at | 1.32 | 0.0083 |
| 1110008H02Rik | RIKEN cDNA 1110008H02 gene | 96156_at | 1.32 | 0.0481 |
| Marcks | Myristoylated alanine rich protein kinase C substrate | 96865_at | 1.32 | 0.0036 |
| Rps28 | ribosomal protein S28 | 100758_at | 1.33 | 0.0013 |
| Stmn2 | stathmin-like 2 | 95670_at | 1.33 | 0.0101 |
| Rps15a | Ribosomal protein S15a | 93730_at | 1.33 | 0.0067 |
| Sox4 | SRY-box containing gene 4 | 160109_at | 1.33 | 0.0044 |
| Nfib | nuclear factor I/B | 99440_at | 1.33 | 0.0274 |
| Rps4x | Ribosomal protein S4, X-linked | 100780_at | 1.33 | 0.0169 |
| Rpl10a | Ribosomal protein L10A | 161327_f_at | 1.33 | 0.0089 |
| Nsep1 | nuclease sensitive element binding protein 1 | 93740_at | 1.34 | 0.0035 |
| Rps19 | ribosomal protein S19 | 94068_at | 1.34 | 0.0006 |
| Dbn1 | drebrin 1 | 103430_at | 1.34 | 0.0021 |
| Cte1 | cytosolic acyl-CoA thioesterase 1 | 103581_at | 1.34 | 0.0417 |
| 2510015F01Rik | RIKEN cDNA 2510015F01 gene | 96885_at | 1.35 | 0.0066 |
| Stmn1 | stathmin 1 | 97909_at | 1.35 | 0.0010 |
| Tubb5 | tubulin, beta 5 | 94788_f_at | 1.35 | 0.0098 |
| Rps27a | ribosomal protein S27a | 104556_at | 1.36 | 0.0015 |
| Islr | Immunoglobulin superfamily containing leucine-rich repeat | 99010_at | 1.36 | 0.0032 |
| Tmsb10 | thymosin, beta 10 | 98129_at | 1.36 | 0.0000 |
| Rps26 | ribosomal protein S26 | 98564_f_at | 1.36 | 0.0093 |
| Rex3 | reduced expression 3 | 93020_at | 1.37 | 0.0075 |
| Nmyc1 | Neuroblastoma myc-related oncogene 1 | 103048_at | 1.37 | 0.0402 |
| Npnt | Nephronectin | 103721_at | 1.37 | 0.0024 |
| St6galnac5 | ST6 (alpha-N-acetyl-neuraminyl-2,3-beta-galactosyl-1,3)-N-acetylgalactosaminide alpha-2,6-sialyltransferase 5 | 92403_at | 1.37 | 0.0061 |
| Ptprz1 | protein tyrosine phosphatase, receptor type Z, polypeptide 1 | 92378_at | 1.37 | 0.0037 |
| Gap43 | Growth associated protein 43 | 102389_s_at | 1.38 | 0.0203 |
| 2610042L04Rik | RIKEN cDNA 2610042L04 gene | 93568_i_at | 1.38 | 0.0209 |
| Mfap2 | Microfibrillar-associated protein 2 | 101095_at | 1.38 | 0.0114 |
| Tubb3 | tubulin, beta 3 | 161612_f_at | 1.38 | 0.0005 |
| 3110003A17Rik | RIKEN cDNA 3110003A17 gene | 96135_at | 1.39 | 0.0041 |
| Arbp | acidic ribosomal phosphoprotein P0 | 101213_at | 1.39 | 0.0022 |
| Igfbp2 | insulin-like growth factor binding protein 2 | 98627_at | 1.39 | 0.0178 |
| Rps12 | ribosomal protein S12 | 102126_at | 1.40 | 0.0088 |
| S100a13 | S100 calcium binding protein A13 | 100959_at | 1.40 | 0.0032 |
| S100a13 | S100 calcium binding protein A13 | 161121_f_at | 1.41 | 0.0058 |
| Hmgn2 | high mobility group nucleosomal binding domain 2 | 101589_at | 1.41 | 0.0016 |
| Hist2h3c2 /// Hist3h2a /// Hist1h2ac /// Hist1h2ad /// Hist1h2ae /// Hist1h2ag /// Hist1h2ah /// Hist1h2ak /// Hist1h2an /// Hist1h2ao /// Hist1h2ab /// Hist1h2ai | histone 2, H3c2 /// histone 3, H2a /// histone 1, H2ac /// histone 1, H2ad /// histone 1, H2ae /// histone 1, H2ag /// histone 1, H2ah /// histone 1, H2ak /// histone 1, H2an /// histone 1, H2ao /// histone 1, H2ab /// histone 1, H2ai | 94805_f_at | 1.42 | 0.0069 |
| Rpl10a | Ribosomal protein L10A | 100711_at | 1.42 | 0.0115 |
| Sh3bgrl | SH3-binding domain glutamic acid-rich protein like | 93806_at | 1.42 | 0.0251 |
| Ppic | Peptidylprolyl isomerase C | 100089_at | 1.43 | 0.0036 |
| Crmp1 | collapsin response mediator protein 1 | 104383_at | 1.43 | 0.0013 |
| Ppp1r14b | protein phosphatase 1, regulatory (inhibitor) subunit 14B | 160078_at | 1.43 | 0.0237 |
| Mki67 | antigen identified by monoclonal antibody Ki 67 | 99457_at | 1.44 | 0.0091 |
| Sox4 | SRY-box containing gene 4 | 101430_at | 1.44 | 0.0325 |
| 2610042L04Rik | RIKEN cDNA 2610042L04 gene | 93569_f_at | 1.45 | 0.0237 |
| Cirbp | cold inducible RNA binding protein | 93284_at | 1.45 | 0.0014 |
| Tnc | tenascin C | 101993_at | 1.47 | 0.0194 |
| Hn1 | hematological and neurological expressed sequence 1 | 93276_at | 1.48 | 0.0085 |
| Mef2c | myocyte enhancer factor 2C | 104590_at | 1.48 | 0.0135 |
| Ednrb | endothelin receptor type B | 103550_at | 1.48 | 0.0076 |
| Clic1 | chloride intracellular channel 1 | 95654_at | 1.50 | 0.0376 |
| Ptprz1 | protein tyrosine phosphatase, receptor type Z, polypeptide 1 | 92380_r_at | 1.58 | 0.0092 |
| Dlx1 | distal-less homeobox 1 | 98394_at | 1.58 | 0.0401 |
| Cks2 | CDC28 protein kinase regulatory subunit 2 | 97527_at | 1.65 | 0.0083 |
| Nnat | neuronatin | 97520_s_at | 1.66 | 0.0217 |
| Hmgb3 | high mobility group box 3 | 98038_at | 1.72 | 0.0004 |
| Dpysl3 | dihydropyrimidinase-like 3 | 101503_at | 1.73 | 0.0339 |
| H19 | H19 fetal liver mRNA | 93028_at | 1.77 | 0.0278 |
| Ptprz1 | protein tyrosine phosphatase, receptor type Z, polypeptide 1 | 92379_f_at | 1.82 | 0.0020 |
| Cd24a | CD24a antigen | 100600_at | 1.84 | 0.0030 |
| Dcx | doublecortin | 102307_at | 1.87 | 0.0085 |
| Sox11 | SRY-box containing gene 11 | 101631_at | 1.88 | 0.0169 |
| Hmgb2 | high mobility group box 2 | 93250_r_at | 1.92 | 0.0004 |
| Rbm3 | RNA binding motif protein 3 | 96041_at | 1.98 | 0.0037 |
| Sox11 | SRY-box containing gene 11 | 93669_f_at | 2.22 | 0.0029 |
| Fabp7 | fatty acid binding protein 7, brain | 98967_at | 2.59 | 0.0002 |

**^1^** in *Nfia^-/-^* mice relative to *Nfia^+/+^* mice. Only probe sets with fold changes of more than 1.20 were considered as representing significantly dysregulated transcripts.

**^2^** only probe sets with p values < 0.05 were considered as representing significantly dysregulated transcripts

**^3^** Some genes (e.g. *Mobp*) were represented by more than one probe set on the microarray (compare results' section). In this table, all individual probe sets are listed with their respective fold change.
